# Benzyl Furanones and Pyrones from the Marine-Derived Fungus *Aspergillus terreus* Induced by Chemical Epigenetic Modification

**DOI:** 10.3390/molecules25173927

**Published:** 2020-08-27

**Authors:** Jing-Shuai Wu, Xiao-Hui Shi, Ya-Hui Zhang, Chang-Lun Shao, Xiu-Mei Fu, Xin Li, Guang-Shan Yao, Chang-Yun Wang

**Affiliations:** 1 Key Laboratory of Marine Drugs, The Ministry of Education of China, School of Medicine and Pharmacy, Institute of Evolution & Marine Biodiversity, Ocean University of China, Qingdao 266003, China; wujingshuai0110@sina.cn (J.-S.W.); 13061461893@163.com (X.-H.S.); 15689932652@163.com (Y.-H.Z.); shaochanglun@163.com (C.-L.S.); fuxiumei92@163.com (X.-M.F.); lixin8962@ouc.edu.cn (X.L.); 2 Laboratory for Marine Drugs and Bioproducts, Qingdao National Laboratory for Marine Science and Technology, Qingdao 266237, China; 3 Institute of Oceanography, Minjiang University, Fuzhou 350108, China

**Keywords:** marine-derived fungus, *Aspergillus terreus*, chemical epigenetic modification, benzyl furanone, benzyl pyrone

## Abstract

Chemical epigenetic modification on a marine-derived fungus *Aspergillus terreus* RA2905 using a histone deacetylase inhibitor, suberoylanilide hydroxamic acid (SAHA), resulted in a significantly changed metabolic profile. A chemical investigation of its ethyl acetate (EtOAc) extract led to the isolation of a racemate of benzyl furanone racemate (±)-**1**, which further separated chirally as a pair of new enantiomers, (+)- and (−)-asperfuranone (**1**), together with two new benzyl pyrones, asperpyranones A (**2**) and B (**3**). Their structures were elucidated by analysis of the comprehensive spectroscopic data, including one-dimensional (1D) and two-dimensional (2D) NMR, and HRESIMS. The absolute configurations were determined by electronic circular dichroism (ECD) calculation and single-crystal X-ray crystallographic experiment. The structures with benzyl furanone or benzyl pyrone skeletons were discovered from natural products for the first time. Compounds (±)-**1**, (+)-**1**, (−)-**1**, and **2** displayed the antifungal activities against *Candida albicans* with MIC values of 32, 16, 64, and 64 μg/mL and PTP1B inhibitory activities with the IC_50_ values of 45.79, 17.32, 35.50, and 42.32 μM, respectively. Compound **2** exhibited antibacterial activity against *Pseudomonas aeruginosa* with the MIC value of 32 μg/mL.

## 1. Introduction

Marine fungi have proven to be a prolific source of structurally novel and biologically active secondary metabolites [1]. Among them, the widespread fungi of *Aspergillus* genus have been extensively investigated for decades due to their chemical diversities and the respected pharmacological activities of secondary metabolites, such as polyketones, xanthones, alkaloids, cyclic peptides, and terpenes [2,3,4]. However, a significant number of genome sequencing analysis of *Aspergillus* species have revealed that most biosynthetic gene clusters are silent or expressed at low levels under standard laboratory conditions, indicating that the capacity of these fungal strains to produce natural products is far more than we anticipated [5]. In recent years, chemical epigenetic manipulation has been widely used as an effective and handy approach to turn on the cryptic biosynthetic pathways in *Aspergillus* species to obtain induced secondary metabolites. For example, chemical epigenetic manipulation was applied to the marine algicolous fungus *Aspergillus terreus* OUCMDZ-2739 with 10 μM histone deacetylase inhibitor, trichostatin A (TSA), resulting in the production of four new meroterpenoids [6].The marine-derived fungus *A. terreus* PF26 was cultured with 900 μM histone deacetylase inhibitor, suberoylanilide hydroxamic acid (SAHA), inducing the enhancement of the production of (+)-terrein [7]. A sea sediment-derived fungus *Aspergillus sydowii* was treated with a DNA methyltransferase inhibitor, 5-azacytidine (5-Aza), leading to the isolation of a series of new bisabolene-type sesquiterpenoids [8]. Therefore, chemical epigenetic manipulation exhibited tremendous potential for excavating cryptic secondary metabolites from marine-derived *Aspergillus* species.

In recent years, during our efforts to explore new bioactive marine natural products [9], chemical epigenetic manipulation has been applied to marine-derived fungi to mine their potential ability to produce metabolic products [9,10,11,12,13,14]. In our previous work, a series of thiodiketopiperazines and 3,4-dihydroisocoumarin derivatives were isolated from the starch liquid culture of *A. terreus* RA2905 derived from sea hare *Aplysia pulmonica* [15]. In the present study, in order to tap the metabolic potential of this fungal strain, chemical epigenetic modifiers were applied to activate its cryptic secondary biosynthetic pathways. The metabolic profile was changed by cultivating with SAHA, detected by HPLC. The chemical investigation of its ethyl acetate (EtOAc) extract resulted in the isolation of a racemate of benzyl furanone, which further separated chirally as a pair of new enantiomers, and two new benzyl pyrones. Herein, we report the epigenetic manipulation on this fungus, and the isolation, structural determination, and bioactivity evaluation of the induced products.

## 2. Results

**Figure 1:**
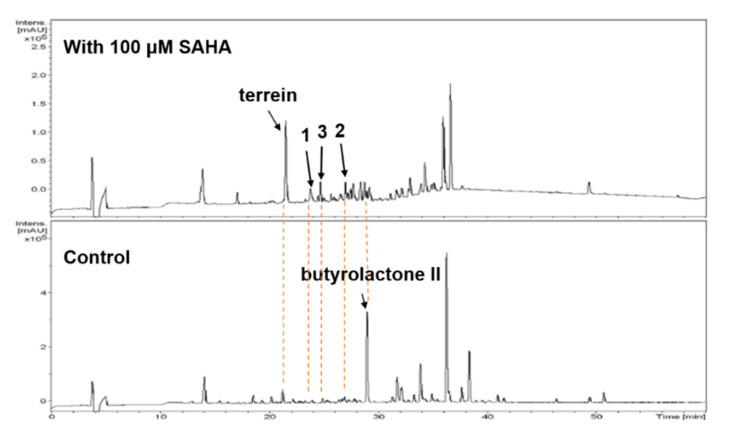
HPLC detection on ethyl acetate (EtOAc) extracts of Aspergillus terreus RA2905 with 100 μM SAHA.

**Figure 2:**
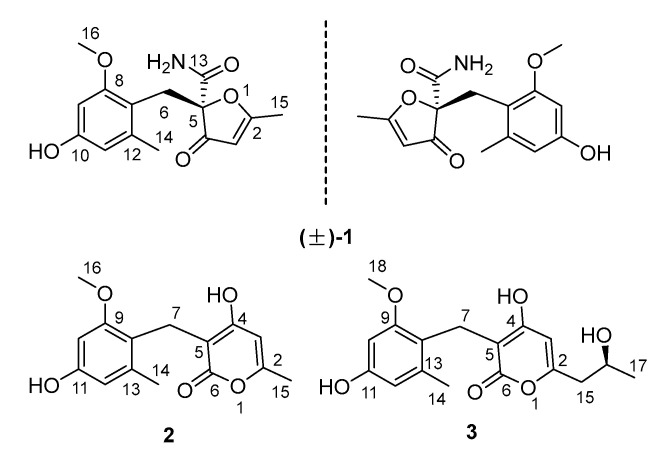
Chemical structures of compounds.

The chemical epigenetic manipulation on *A. terreus* RA2905 was conducted in a starch liquid medium with the conditions of 180 rpm, 28 °C, for 7 days, cultivated with histone deacetylase inhibitors and DNA methyltransferase inhibitors in different concentrations (1−1000 μM). The fungal culture in the same medium without an inhibitor was used as the control. As a result, in the HPLC profiles, much more remarkable peaks (20−30 min) emerged in the fungal culture treated with 100 μM SAHA as compared with the control (Figure 1). The production of terrein was significantly increased about nine-fold, while the production of butyrolactones was decreased, especially butyrolactone II. Subsequently, a scaled-up fermentation with 100 μM SAHA was carried out. The EtOAc extract of the culture was separated by using column chromatography, semi-preparative and chiral HPLC, resulting in the isolation of a racemate of benzyl furanone racemate, (±)-**1**, which further separated chirally as a pair of new enantiomers, (+)- and (−)-asperfuranone (**1**), and two benzyl pyrones, asperpyranones A (**2**) and B (**3**) (Figure 2).

### 2.1. Structure Elucidation

**Figure 3:**
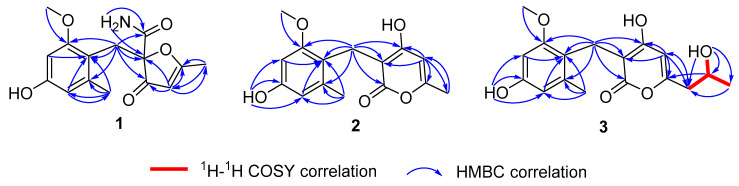
The ^1^H−^1^H COSY and key HMBC correlations of **1** and **3**.

Compound **1** was obtained as a white crystal. The molecular formula of C_15_H_17_O_5_N was deduced by HRESIMS spectrum, indicating eight degrees of unsaturation. The stretch signals at 3300, 2892, 2845, 2350, 1709, 1607, 1452, 1385, and 1025 cm^−1^ in the IR spectrum suggested the presence of hydroxyl/amino, carbonyl, and aromatic groups in **1**. The ^1^H NMR spectrum showed the proton signals for 1,2,3,5-tetrasubstituted benzene ring (*δ*_H_ 6.14, d, *J* = 2.3 Hz and 6.12, d, *J* = 2.3 Hz), three active hydrogen signals (*δ*_H_ 9.21, 7.31, and 7.23), one hydrogen signal for double bond (*δ*_H_ 5.32), one methoxyl (*δ*_H_ 3.57), one methylene (*δ*_H_ 3.25 and 3.19), and two methyl (*δ*_H_ 2.11 and 2.02) (Table 1). A total of 15 carbon atoms were observed in the ^13^C NMR spectrum, including three methyls (one oxygenated), one methylene, one quaternary carbon, eight olefinic carbons (three protonated and two oxygenated), and two carbonyls, as distinguished by the HSQC spectrum. The HMBC correlations from H-6 to C-8/C-12, from H-16 to C-8, and from H-14 to C-7/C-11 revealed the structure of a benzyl group, combining with spectroscopic characteristics of the 1,2,3,5-tetrasubstituted benzene ring (Figure 3). In addition, a spin system of C-6−C-5−C-4−C-3−C-2−C-15 was established according to the HMBC correlations from H-3 to C-2/C-4/C-5/C-15, from H-6 to C-4/C-5/C-13, and from H-15 to C-2/C-3. As the presence of two carbonyl groups, one aromatic ring and one double bond in the molecule occupied seven degrees of unsaturation, compound **1** should contain one furan ring formed by C-2 and C-5 in consideration of their carbon chemical shifts (*δ*_C_ 189.8 for C-2, *δ*_C_ 91.4 for C-5). The HMBC correlations from H-6 to C-13 and 13-NH_2_ to C-5/C-13 indicated that the NH_2_ group was anchored to the carbonyl carbon, C-13, and this carbonyl was located at the quaternary carbon, C-5. Thus, the planar structure of **1** was established.

**Figure 4:**
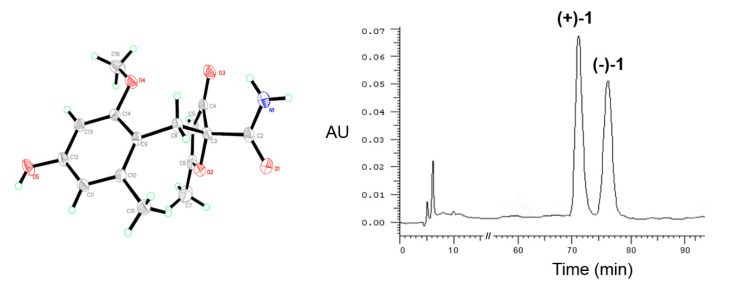
X-ray ORTEP drawing of **1** and chiral HPLC chromatogram of (±)-**1**.

After many attempts, a suitable crystal of **1** was obtained by evaporating slowly in a mixed solvent of CH_2_Cl_2_/acetone/MeOH (1:1:1). However, the space group of C2/c of its crystal, combined with its optical value of 0, compound **1** was suspected to be a racemate. Then, by using a chiral chromatographic column, a pair of enantiomers, (+)- and (−)-**1**, were separated with the peak area ratio of 1:1 (Figure 4).

**Figure 5:**
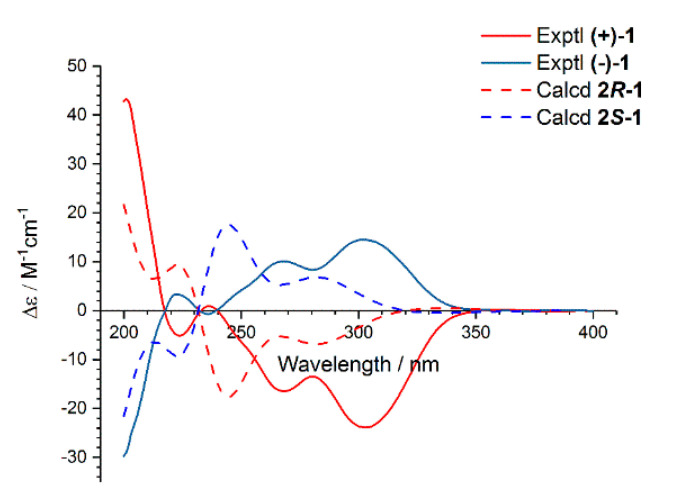
Experimental and calculated electronic circular dichroism (ECD) spectra of (+)-**1** and (−)-**1** in MeOH.

In order to determine the absolute configurations of (+)- and (−)-**1**, the calculations for their electronic circular dichroism (ECD) spectra were performed by using the time-dependent density functional theory (TDDFT) at the B3LYP/6-311+G(d,p) level in MeOH (Table S1). The results showed that the calculated ECD spectra of (+)- and (−)-**1** were in accordance with their experimental spectra with negative and positive Cotton effects in the range of 240−340 nm, respectively (Figure 5). Thus, (+)- and (−)-**1** were defined with absolute configurations as 2*R* and 2*S*, and named as (+)- and (−)-asperfuranone, respectively.

Compound **2** was determined to be C_15_H_16_O_5_ with eight degrees of unsaturation on the basis of HRESIMS data. By careful analysis of its ^1^H and ^13^C NMR data, the spectroscopic characteristics of the benzyl group (from C-7 to C-13) in **2** were revealed, which were the same as those of compound **1** (Table 2). One methoxyl and one methyl were located at C-9 and C-13 of the benzyl group, respectively, based on the HMBC correlations from H-16 to C-9, and from H-14 to C-8/C-12/C-13. A spin system of C-15−C-2−C-3−C-4−C-5−C-6 was established base on the HMBC correlations from H-3 to C-2/C-4/C-5/C-15 and from H-15 to C-2/C-3 (Figure 3). As the presence of one carbonyl group, one aromatic ring and two double bonds in the molecule occupied seven degrees of unsaturation, compound **2** should contain one pyran ring formed between C-2 and C-6 in consideration of their carbon chemical shifts (*δ*_C_ 164.9 for C-2, *δ*_C_ 164.3 for C-6). The HMBC correlations from H-7 to C-4/C-5/C-6 suggested that the benzyl group was anchored to C-5 of the pyran ring. One methyl was connected to C-6 in the pyran ring based on the HMBC correlations from H-3 to C-15 and from H-15 to C-3. Thus, the structure of **2** was established, and named as asperpyranone A.

**Figure 6:**
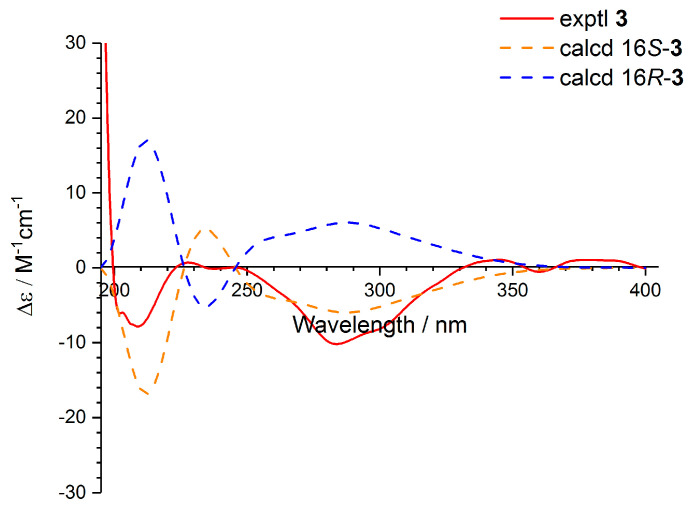
Experimental and calculated ECD spectra of **3** in MeOH.

Compound **3** was given as C_17_H_20_O_6_ with eight degrees of unsaturation based on the HRESIMS data. The ^1^H and ^13^C NMR spectroscopic characteristics of **3** were similar to those of **2**, which revealed the presence of benzyl pyranone structure (Table 2). The main difference was that the methyl (*δ*_H_ 2.10, *δ*_C_ 19.2) in **2** was replaced by an isopropanol group (*δ*_H_ 2.39, *δ*_C_ 42.7 for CH_2_-15; *δ*_H_ 3.87, *δ*_C_ 64.1 for CH-16; *δ*_H_ 1.07, *δ*_C_ 23.4 for CH_3_-17) in **3**, which was verified by the corresponding ^1^H−^1^H COSY of H-15/H-16/H-17 and H-16/16-OH, and HMBC correlations from 16-OH to C-15/C-16/C-17 and from H-17 to C-15 (Figure 3). The isopropanol was located at C-2 based on the HMBC correlations from H-3 to C-15, from H-15 to C-3, and from H-16 to C-2. At first, an unsuccessful attempt was made to assign the absolute configuration of C-16 by Mosher’s method, possibly due to the influence of hydroxyl groups on the C-11 of aromatic ring or C-4 of double bond in **3**. Furthermore, the calculation of ECD of 16*S*-**3** and 16*R*-**3** was performed (Table S1). The results showed that the experimental ECD spectrum was in accordance with the calculated spectrum of 16*S*-**3** with the negative Cotton effects at 210 and 280 nm (Figure 6). Thus, compound **3** was tentatively assigned with the absolute configuration of 16*S*, and named as asperpyranone B.

**Figure 7:**
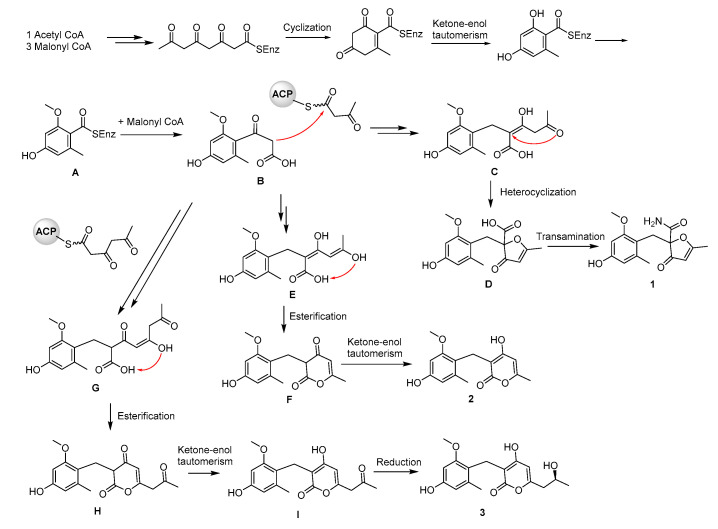
The biosynthetic pathway of benzyl furanone (**1**) and pyrones (**2** and **3**).

The benzyl furanone and pyrone metabolites were supposed to be produced in this strain through the fungal PKS biosynthetic pathway activated by epigenetic modification (Figure 7). The structure of benzyl, intermediate A, was synthesized with condensation and cyclization reactions from one acetyl-CoA and three malonyl-CoA. One malonyl-CoA was added into the intermediate A to produce intermediate B, which was further fused with two malonyl-CoA to afford intermediate C through Claisen condensation, reduction, and rearrangement reactions. Heterocyclization along with double-bond isomerizations provided the intermediate D with the furanone ring [16]. A NH_2_ group was reacted with carboxyl to give compound **1**. The intermediate B fused with two malonyl-CoA to give intermediate E. When the hydroxyl group on the terminal double bond of intermediate E was condensed with the carboxyl, the pyrone ring was formed to yield intermediate F. The following ketone-enol tautomerization afforded compound **2**. While two malonyl-CoA were added into the intermediate B resulting in the production of intermediate G with Claisen condensation, reduction, and rearrangement reactions. Similarly, the condensation between hydroxyl on the terminal double bond and the carboxyl on intermediate G, and the subsequent ketone-enol tautomerization formed intermediate I. The carbonyl of intermediate I was reduced to hydroxyl to yield compound **3**.

### 2.2. Bioassays

All of the isolated compounds were tested for their antifungal, antibacterial, cytotoxic, and PTP1B inhibitory activities (Table S2). Compounds (±)-**1**, (+)-**1**, (−)-**1**, and **2** displayed the antifungal activities against *Candida albicans* with the MIC values of 32, 16, 64, and 64 μg/mL (0.5 μg/mL for positive control amphotericin B). Compounds **2** and **3** exhibited antibacterial activities against *Pseudomonas aeruginosa* ATCC 27853 with the MIC values of 32 and 128 μg/mL, respectively (1 μg/mL for positive control vancomycin). Compounds (±)-**1**, (+)-**1**, (−)-**1**, and **2** also showed PTP1B inhibitory activities with the IC_50_ values of 45.79, 17.32, 35.50, and 42.32 μM, respectively (4 μM for positive control oleanolic acid). All of the tested compounds showed no cytotoxicity. Interestingly, the isomers (+)-**1** and (−)-**1** displayed differentiated effects, of which (+)-**1** exhibited more active than (−)-**1** in antifungal and PTP1B inhibitory activities.

## 3. Materials and Methods

### 3.1. Instrumentation

Optical rotations were measured on a JASCO P-1020 digital polarimeter (Jasco Corp., Tokyo, Japan). UV spectra were recorded on a HITACHI UH 5300 UV spectrophotometer (Hitachi, Tokyo, Japan). The ECD data were acquired on a J-815-150S Circular Dichroism spectrometer (JASCO Electric Co., Ltd., Tokyo, Japan). The IR spectra were recorded on a Nicolet-Nexus-470 spectrometer (Thermo Electron Co., Madison, WI, USA) using KBr pellets. The NMR spectra were acquired by a JEOL JEM-ECP NMR spectrometer (500 MHz for ^1^H and 125 MHz for ^13^C, JEOL, Tokyo, Japan), using TMS as an internal standard. HREIMS were measured on a Thermo MAT95XP high resolution mass spectrometer (Thermo Fisher Scientific, Bremen, Germany), and EIMS spectra on a Thermo DSQ EImass spectrometer (Thermo Fisher Scientific, Bremen, Germany). Single-crystal X-ray crystallographic analysis was performed on an Agilent Xcalibur Eos Gemini diffractometer (Agilent Technologies, Yarnton, England). Samples were analyzed on a Hitachi L-2000 HPLC system coupled with a Hitachi L-2455 photodiode array detector and using a C_18_ column (Kromasil 250 × 4.6 mm, 5 μm). The semi-prepared HPLC was conducted by a semi-prepared C_18_ column (Kromasil 250 × 10 mm, 5 μm). The chiral HPLC chromatographic isolation of compound **1** was conducted using the semi-prepared 5-TBB column (Kromasil 250 × 10 mm, 5 μm). Silica gel (Qing Dao Hai Yang Chemical Group Co., China; 300−400 mesh) and Sephadex LH-20 (Amersham Biosciences) were used for column chromatography (CC). Precoated silica gel plates (Yan Tai Zi Fu Chemical Group Co., China; G60, F-254) were used for thin-layer chromatography. PTP1B (human recombinant) was purchased from Abcam (ab51277).

### 3.2. Fungal Material

The fungal strain was isolated from a piece of fresh tissue from the inner part of the sea hare *Aplysia pulmonica*, collected from the Weizhou coral reefs in the South China Sea during April 2010. The fungus was identified as *Aspergillus terreus* based on the sequence analysis (GenBank accession no. MK611650) of the ITS region of the rRNA gene, as described previously [15]. This fungal strain was deposited in the Key Laboratory of Marine Drugs, Ministry of Education of China, School of Medicine and Pharmacy, Ocean University of China, Qingdao, China.

### 3.3. Extraction and Isolation

Sixty 500 mL Erlenmeyer flasks of the fungal strain were cultivated in the starch liquid medium (soluble starch 10 g/L, peptone 1 g/L, artificial sea salt 30 g/L, 200 mL each flask) by adding 100 μM SAHA with the conditions of 150 rpm, 28 °C, for 7 days. The fermentation broth and mycelia were separated through cheesecloth and extracted repeatedly with equal amounts of ethyl acetate (EtOAc) for three times, respectively. The extracts were combined and evaporated in vacuo to afford an EtOAc extract (11.2 g). The EtOAc extract was isolated on silica gel column chromatography (CC) using a step gradient elution with petroleum ether/acetone (10:1 to 1:4, *v*/*v*) to provide six fractions (Fr.1−Fr.6). Fr.4 was separated by Sephadex LH-20 CC eluted with CH_2_Cl_2_/MeOH (1:1, *v*/*v*) and the semi-preparative HPLC with MeOH/H_2_O (55:45, *v*/*v*) to give compounds **1** (4.2 mg), **2** (3.5 mg), and **3** (1.9 mg). Compound **1** was subjected to the chiral chromatographic HPLC with isopropanol/ n-hexane (10:90, *v*/*v*) as the elution system to provide (+)- **1** (1.2 mg) and (−)-**1** (0.9 mg).

*Asperfuranone* (**1**). white crystal; ${\left[\alpha \right]}_{D}^{20}$ 0 (*c* 0.5, MeOH); UV (MeOH) *λ*_max_ (log *ε*) 221 (1.41), 249 (0.63) nm; IR (KBr) *ν*_max_ 3300, 2892, 2845, 2350, 1709, 1607, 1452, 1385, 1025 cm^−1^; ^1^H NMR (500 MHz, DMSO-*d*_6_), and ^13^C NMR (125 MHz, DMSO-*d*_6_), see Table 1; HRESIMS *m*/*z* 292.1182 [M + H]^+^ (calcd for C_15_H_18_O_5_N, 292.1179), *m*/*z* 314.0998 [M + Na]^+^ (calcd for C_15_H_17_O_5_NNa, 314.0999).

Crystal data for **1**: C_15_H_17_O_5_N, *M*r = 291.11, monoclinic, *a* = 14.5365(4) Å, *b* = 11.9873(2) Å, *c* = 17.8891(4) Å, *α* = 90.00°, *β* = 115.707(3)°, *γ* = 90.00°, *V* = 2808.72(12) Å^3^, space group *C2*/*c*, Z = 8, *D*x = 1.378 mg/m^3^, *μ* (Cu Kα) = 0.896 mm^–1^, and *F* (000) = 1232. Crystal dimensions: 0.08 × 0.07 × 0.07 mm^3^. Independent reflections: 4781/2518 (*R*_int_ = 0.0142). The final R1 value was 0.0386, *wR*2 = 0.1019 (*I* > 2*σ(I)*). Crystallographic data of **1** have been deposited in the Cambridge Crystallographic Data Centre (deposition number CCDC 1984035).

*(+)-Asperfuranone* (**1**). ${\left[\alpha \right]}_{D}^{20}$ +178.3 (*c* 0.5, MeOH); ECD (MeOH) *λ*_max_ (Δ*ε*) 264 (−10.3), 301 (−13.5).

*(−)-Asperfuranone* (**1**). ${\left[\alpha \right]}_{D}^{20}$ −178.3 (*c* 0.5, MeOH); ECD (MeOH) *λ*_max_ (Δ*ε*) 264 (10.3), 301 (13.5).

*Asperpyranone A* (**2**). yellow solid; UV (MeOH) *λ*_max_ (log *ε*) 220 (1.75), 271 (1.01) nm; IR (KBr) *ν*_max_ 3648, 2358, 1734, 1681, 1651, 1540, 1393, 1203, 1093 cm^−1^; ^1^H NMR (500 MHz, DMSO-*d*_6_) and ^13^C NMR (125 MHz, DMSO-*d*_6_), see Table 2; HRESIMS *m*/*z* 275.0927 [M − H]^−^ (calcd for C_15_H_15_O_5_, 275.0925).

*Asperpyranone B* (**3**). yellow solid; ${\left[\alpha \right]}_{D}^{20}$ +8.1 (*c* 0.5, MeOH); UV (MeOH) *λ*_max_ (log *ε*) 220 (1.95), 263 (0.97) nm; IR (KBr) *ν*_max_ 3748, 3650, 2360, 1734, 1684, 1651, 1540, 1510, 1208, 1140 cm^−1^; ^1^H NMR (500 MHz, DMSO-*d*_6_) and ^13^C NMR (125 MHz, DMSO-*d*_6_), see Table 2; HRESIMS *m*/*z* 321.1333 [M + H]^+^ (calcd for C_17_H_21_O_6_, 321.1333), *m*/*z* 343.1152 [M + Na]^+^ (calcd for C_17_H_20_O_6_Na, 343.1152).

### 3.4. ECD Calculation for Compounds *(+)-**1***, *(−)-**1***, and ***3***

Monte Carlo conformational searches were carried out by means of the Spartan’s 10 software using Merck Molecular Force Field (MMFF). The conformers with Boltzmann-population of over 5% were chosen for NMR calculations, and then the conformers were initially optimized at B3LYP/6-31G(d,p) level in MeOH using the CPCM polarizable conductor calculation model. The theoretical calculation of ECD was performed in MeOH using time-dependent density functional theory (TD-DFT) at the B3LYP/6-311+G(d,p) level for all conformers of compounds (+)-**1**, (−)-**1**, and **3**, respectively. Rotatory strengths for a total of 50 excited states were calculated. ECD spectra were generated using the program SpecDis 1.6 (University of Würzburg, Würzburg, Germany) from dipole-length rotational strengths by applying Gaussian band shapes with sigma = 0.3 eV.

### 3.5. Antimicrobial Assays

The antibacterial and antifungal assays were conducted in 96-well plates using a broth micro dilution method [17]. Six pathogenic bacterial strains were used for antibacterial activity, including *Staphylococcus epidermidis* ATCC 12228, *Staphylococcus aureus* ATCC 25923, *Pseudomonas aeruginosa* ATCC 27853, *Bacillus cereus* ATCC 14579, *Escherichia coli* ATCC 25922, *and Sarcina lutea* ATCC 9341. Vancomycin was used as a positive control. Three fungal strains *Candida albicans* ATCC 10231, *Candida tropicalis* ATCC 20962, and *Candida parapsilosis* ATCC 22019 were used for antifungal activity. Amphotericin B was used as a positive control.

### 3.6. PTP1B Inhibition Assays

The PTP1B inhibition assay was performed in 96-well plates [18]. The compound (10 μL) was added to the 99 μL reaction buffer solution, which consisted of 10 mM Tris (pH 7.4), 50 mM NaCl, 2 mM dithiothreitol (DTT), 1 mM MnCl_2_, and 10 mM para-nitrophenyl phosphate (*p*NPP). The reaction mix was pre-warmed using a block heater at 37 °C. The recombinant PTP1B solution (1 mg/mL, 1 μL) was mixed in each well. An NaOH solution (10 μL, 0.1 M) was added to stop the reaction. The absorbance was recorded at 405 nm using a microplate. Oleanolic acid was used as a positive control.

### 3.7. Cytotoxicity Assays

The cytotoxic activities were evaluated by the SRB method [19] using five human tumor cell lines A549, HCT116, MCF-7, Hela, and Hep G2. Adriamycin was used as a positive control.

## 4. Conclusions

In summary, the sea hare derived fungus *A. terreus* RA2905 was effectively induced by chemical epigenetic manipulation with histone deacetylase inhibitor, SAHA, resulting in the isolation of a racemate of benzyl furanone racemate, which further separated chirally as a pair of new enantiomers, and two new benzyl pyrones. The harvested products with the structural features with benzyl furanone or benzyl pyrone skeletons were discovered from natural products for the first time. On the basis of our case, it could be concluded that chemical epigenetic manipulation should be a feasible strategy to activate the silent metabolic pathway and tap new secondary metabolites from marine-derived fungi.

## Figures and Tables

**Table 1:** ^1^H NMR (500 MHz) and ^13^C NMR (125 MHz) data for **1** in DMSO-*d*_6_.

Position	*δ*_C_, Type	*δ*_H_ (*J* in Hz)
2	189.8, C	
3	102.0, CH	5.32, s
4	198.9, C	
5	91.4, C	
6	30.1, CH_2_	3.25, d (14.2)
		3.19, d (14.2)
7	111.7, C	
8	159.2, C	
9	96.3, CH	6.14, d (2.3)
10	156.7, C	
11	108.8, CH	6.12, d (2.3)
12	139.3, C	
13	166.7, C	
14	20.0, CH_3_	2.11, s
15	16.2, CH_3_	2.02, s
16	55.0, CH_3_	3.57, s
10-OH		9.21, s
13-NH_2_		7.23, s
		7.31, s

**Table 2:** ^1^H NMR (500 MHz) and ^13^C NMR (125 MHz) data for **2** and **3** in DMSO-*d*_6_.

Position	2		3	
	*δ*_C_, Type	*δ*_H_ (*J* in Hz)	*δ*_C_, Type	*δ*_H_ (*J* in Hz)
2	164.9, C		160.4, C	
3	100.1, CH	5.94, s	100.9, CH	5.96, s
4	159.3, C		164.4, C	
5	100.5, C		100.8, C	
6	164.3, C		164.7, C	
7	19.6, CH_2_	3.46, brs	19.6, CH_2_	3.46, brs
8	117.0, C		117.0, C	
9	158.6, C		158.6, C	
10	96.8, CH	6.15, d (2.3)	96.8, CH	6.16, d (2.3)
11	155.7, C		155.7, C	
12	108.9, CH	6.10, d (2.3)	108.9, CH	6.10, d (2.3)
13	138.1, C		138.1, C	
14	19.9, CH_3_	2.17, s	19.9, CH_3_	2.16, s
15	19.2, CH_3_	2.10, s	42.7, CH_2_	2.39, d (6.4)
16	55.3, CH_3_	3.61, s	64.1, CH	3.87, m
17			23.4, CH_3_	1.07, d (6.1)
18			55.4, CH_3_	3.61, s
OH-11		9.03, s		9.03, s
OH-16				4.77, d (5.1)

## Supplementary Materials

The followings are available online. Figures S1−S20: ^1^H NMR, ^13^C NMR, DEPT, HSQC, ^1^H−^1^H COSY, HMBC, and HRESIMS spectra of compounds **1**−**3**, Table S1: The ECD calculation results of (+)-**1**, (−)-**1**, and **3**, Table S2: The bioactivity assays of isolated compounds.
