# Supplementary material for: Benzyl Furanones and Pyrones from the Marine-Derived Fungus Aspergillus terreus Induced by Chemical Epigenetic Modification
Source: Molecules. 2020 Aug 27;25(17):3927. doi: 10.3390/molecules25173927 (PMC7503933; doi:10.3390/molecules25173927)
Supplement: Supplementary file 1 [file molecules-25-03927-s001.pdf]

Benzyl furanones and pyrones from the marine-derived fungus *Aspergillus terreus* induced by chemical epigenetic modification

Jing-Shuai Wu[1,2], Xiao-Hui Shi[1,2], Ya-Hui Zhang[1,2], Chang-Lun Shao[1,2], Xiu-Mei Fu[1,2], Xin Li[1,2], Guang-Shan Yao[1,2,3,*] and Chang-Yun Wang[1,2,*]

[1] Key Laboratory of Marine Drugs, The Ministry of Education of China, School of Medicine and Pharmacy; Institute of Evolution & Marine Biodiversity, Ocean University of China, Qingdao 266003, People's Republic of China; wujingshuai0110@sina.cn (J.S. Wu); 13061461893@163.com (X.H. Shi); 15689932652@163.com (Y.H. Zhang); shaochanglun@163.com (C.L. Shao); fuxiumei92@163.com (X.M. Fu); lixin8962@ouc.edu.cn (X. Li).

[2] Laboratory for Marine Drugs and Bioproducts, Qingdao National Laboratory for Marine Science and Technology, Qingdao 266237, People's Republic of China.

[3] Institute of Oceanography, Minjiang University, Fuzhou 350108, People's Republic of China.

* Correspondence: changyun@ouc.edu.cn (C.Y. Wang.); +86-053282031536 (C.Y. Wang); ygshan@126.com (G.-S. Yao); +86-059183789471 (G.-S. Yao).

List of supporting information

Figure S1. ^{1}H NMR (500 MHz, DMSO-d_6) spectrum of (±)-**1**

Figure S2. ^{13}C NMR (125 MHz, DMSO-d_6) spectrum of (±)-**1**

Figure S3. HSQC (DMSO-d_6) spectrum of (±)-**1**

Figure S4. ^{1}H–^{1}H COSY (DMSO-d_6) spectrum of (±)-**1**

Figure S5. HMBC (DMSO-d_6) spectrum of (±)-**1**

Figure S6. HRESIMS spectrum of (±)-**1**

Figure S7. ^{1}H NMR (500 MHz, DMSO-d_6) spectrum of compound **2**

Figure S8. ^{13}C NMR (125 MHz, DMSO-d_6) spectrum of compound **2**

Figure S9. DEPT (125 MHz, DMSO-d_6) spectrum of compound **2**

Figure S10. HSQC (DMSO-d_6) spectrum of compound **2**

Figure S11. ^{1}H–^{1}H COSY (DMSO-d_6) spectrum of compound **2**

Figure S12. HMBC (DMSO-d_6) spectrum of compound **2**

Figure S13. HRESIMS spectrum of compound **2**

Figure S14. ^{1}H NMR (500 MHz, DMSO-d_6) spectrum of compound **3**

Figure S15. ^{13}C NMR (125 MHz, DMSO-d_6) spectrum of compound **3**

Figure S16. DEPT (125 MHz, DMSO-d_6) spectrum of compound **3**

Figure S17. HSQC (DMSO-d_6) spectrum of compound **3**

Figure S18. ^{1}H–^{1}H COSY (DMSO-d_6) spectrum of compound **3**

Figure S29. HMBC (DMSO-d_6) spectrum of compound **3**

Figure S20. HRESIMS spectrum of compound **3**

Table S1. The ECD calculation results of (+)-**1**, (−)-**1** and **3**

Table S2. The bioactivity assays of isolated compounds

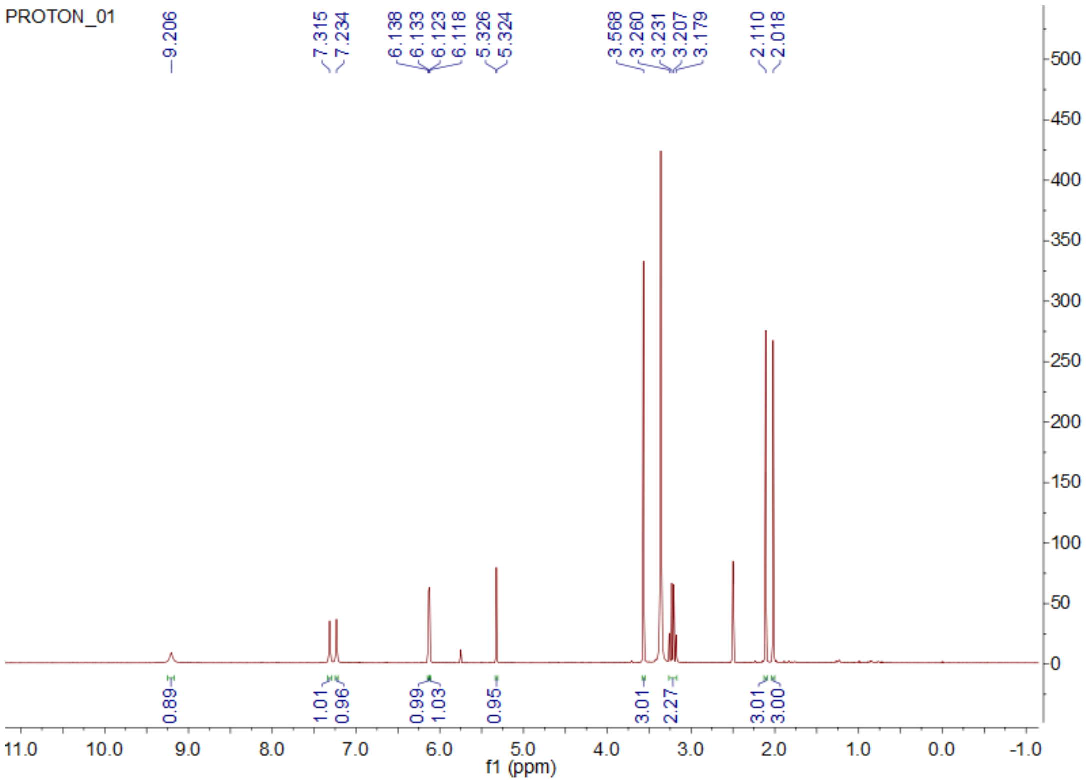

Figure S1. ^{1}H NMR (500 MHz, DMSO-d_6) spectrum of (±)-**1**

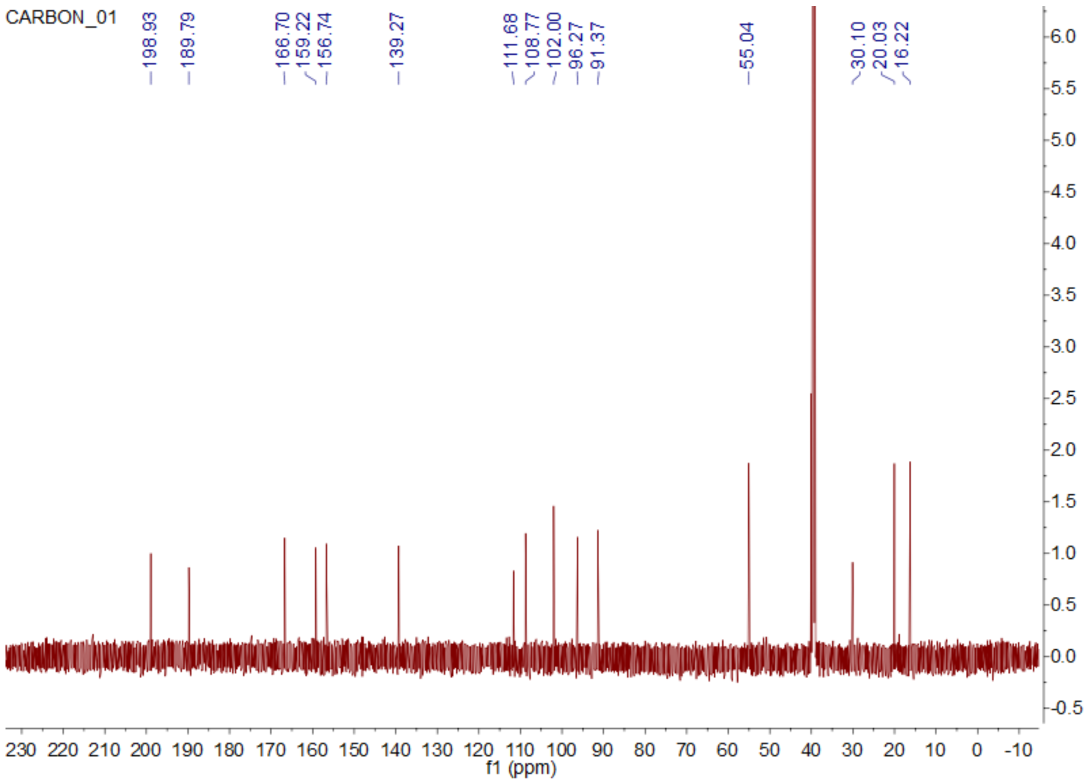

Figure S2. ^{13}C NMR (125 MHz, DMSO-d_6) spectrum of (±)-**1**

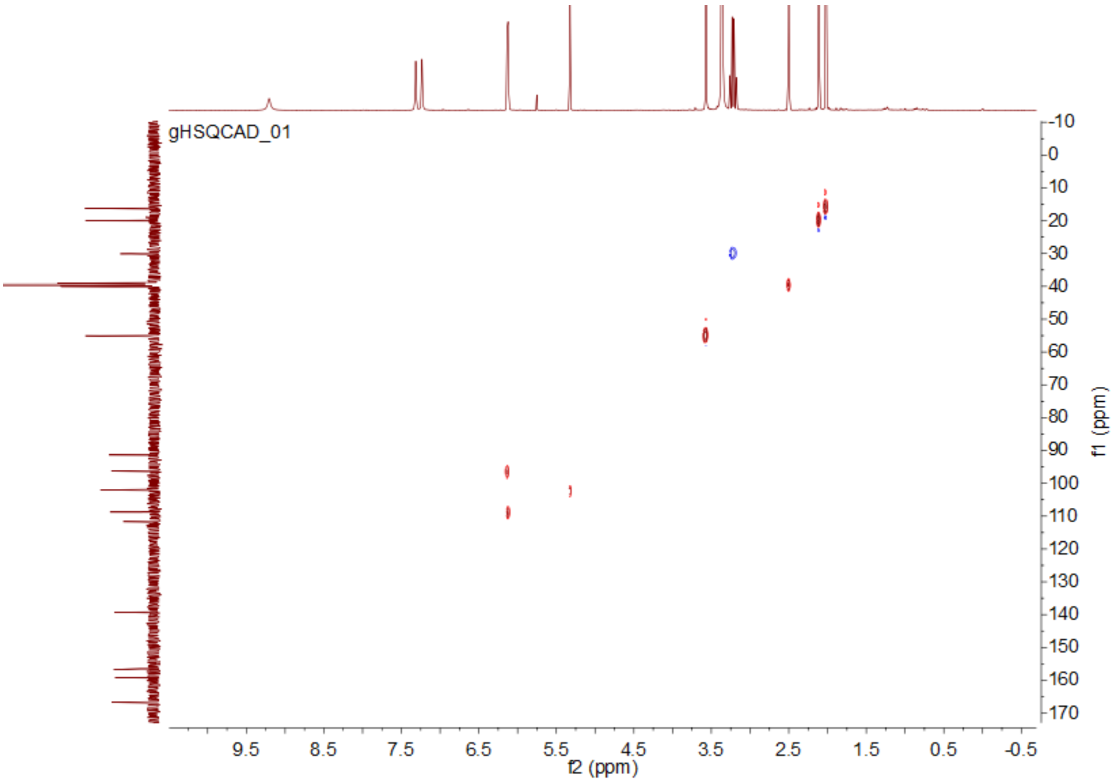

Figure S3. HSQC (DMSO-d_6) spectrum of (±)-**1**

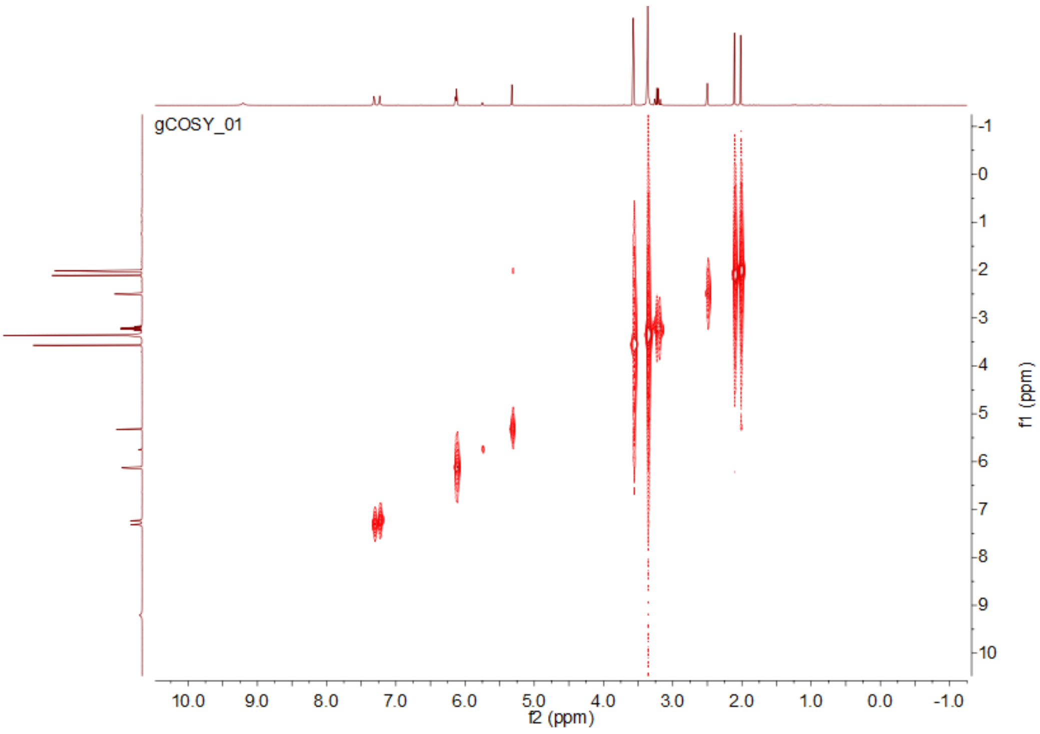

Figure S4. ^{1}H–^{1}H COSY (DMSO-d_6) spectrum of (±)-**1**

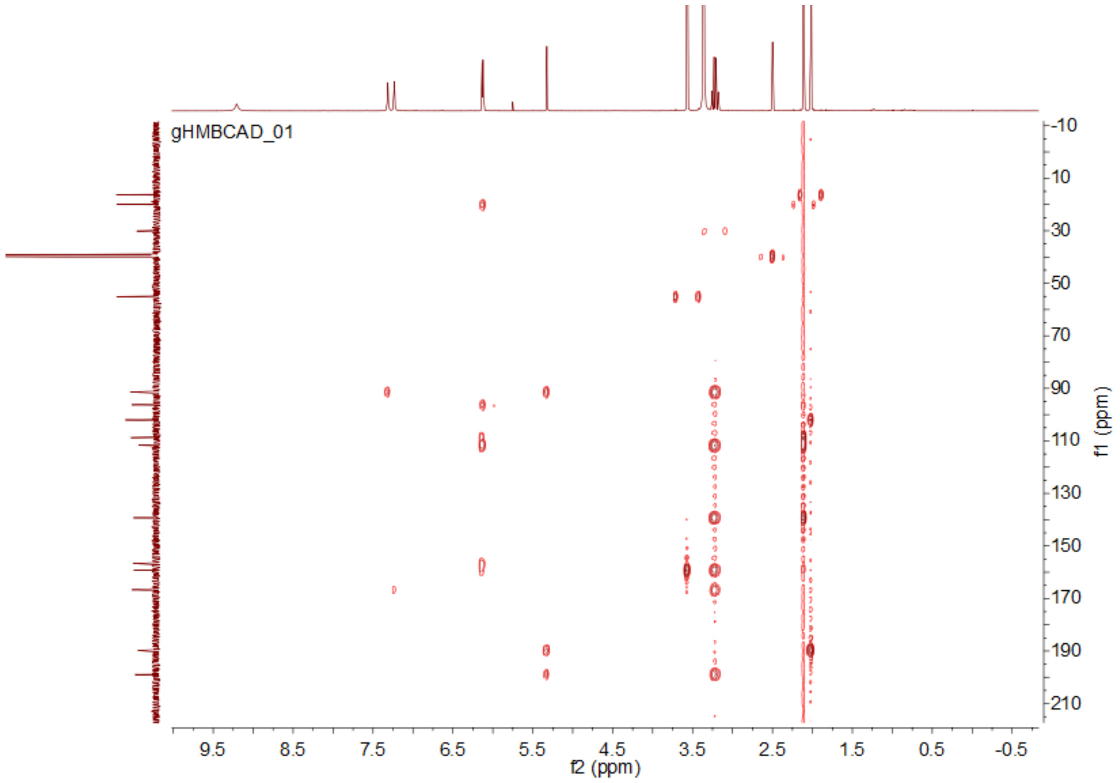

Figure S5. HMBC (DMSO-d_6) spectrum of (±)-**1**

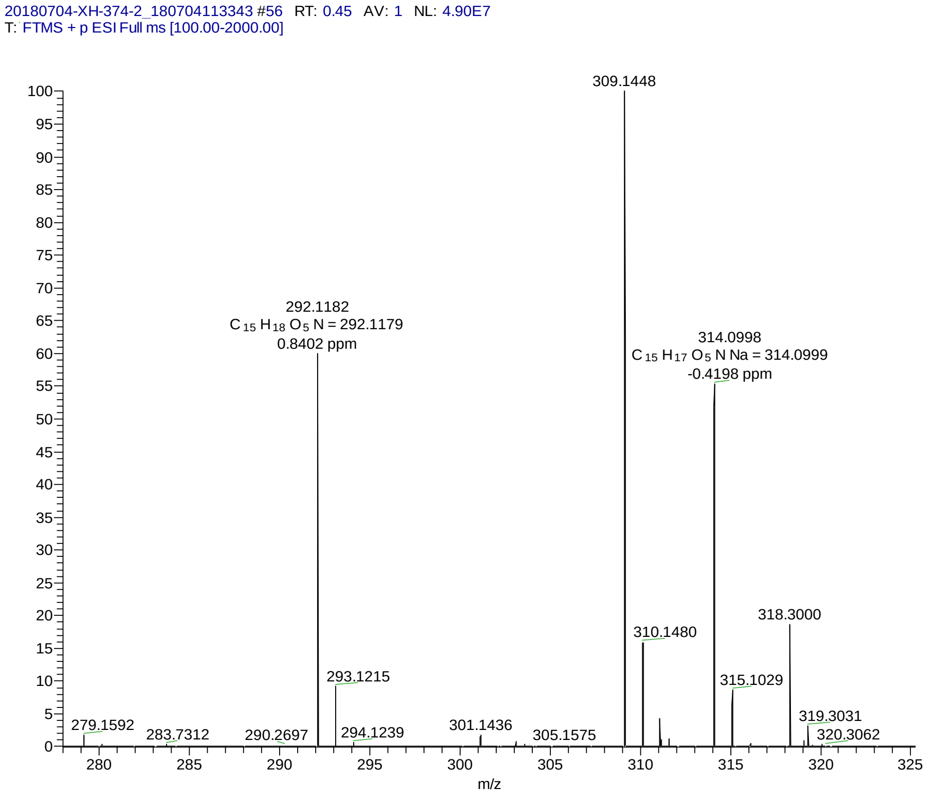

Figure S6. HRESIMS spectrum of (±)-**1**

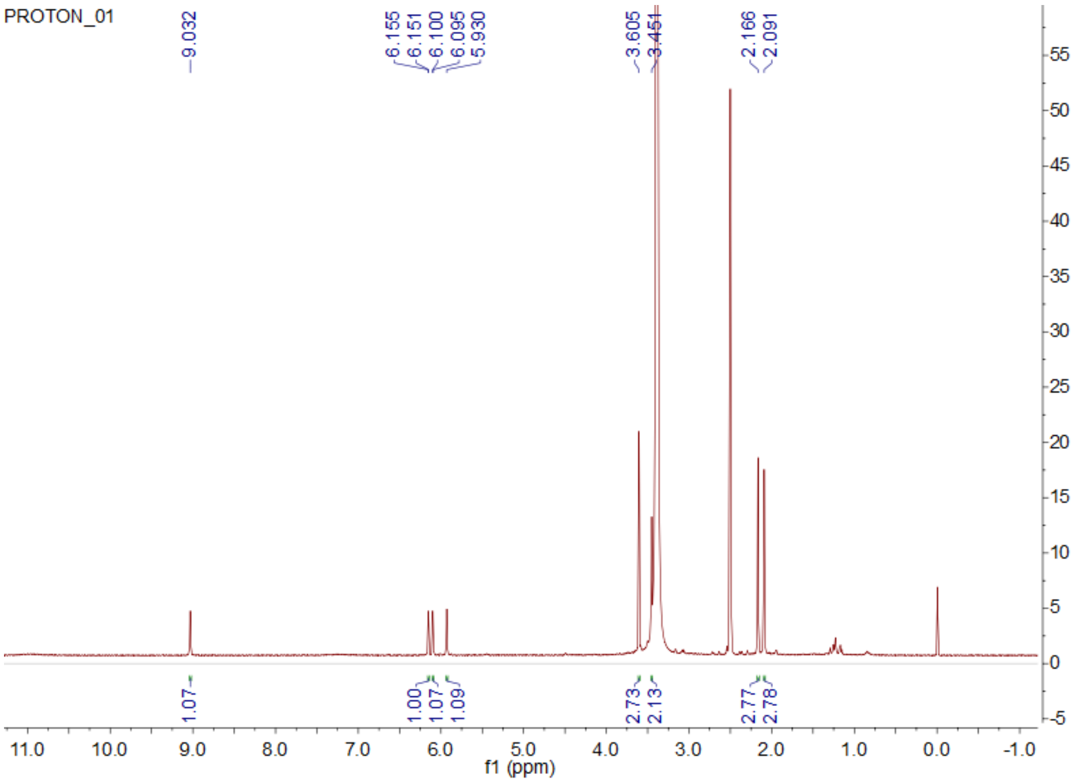

Figure S7. ^{1}H NMR (500 MHz, DMSO-d_6) spectrum of compound **2**

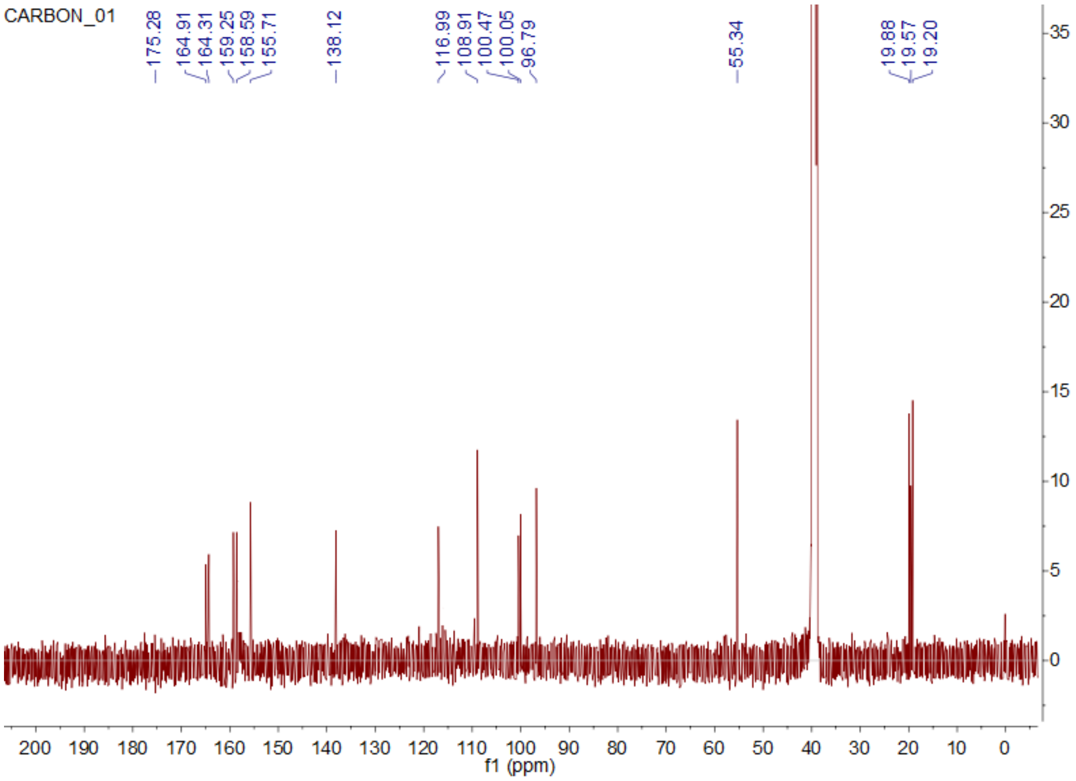

Figure S8. ^{13}C NMR (125 MHz, DMSO-d_6) spectrum of compound **2**

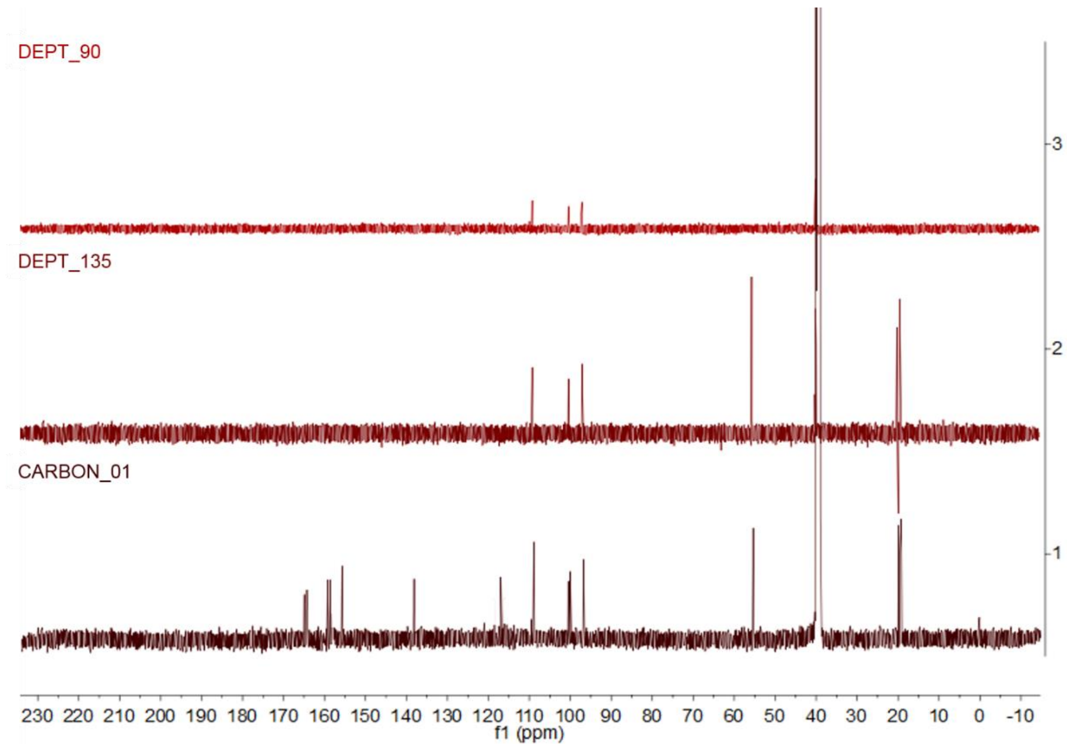

Figure S9. DEPT (125 MHz, DMSO-d_6) spectrum of compound **2**

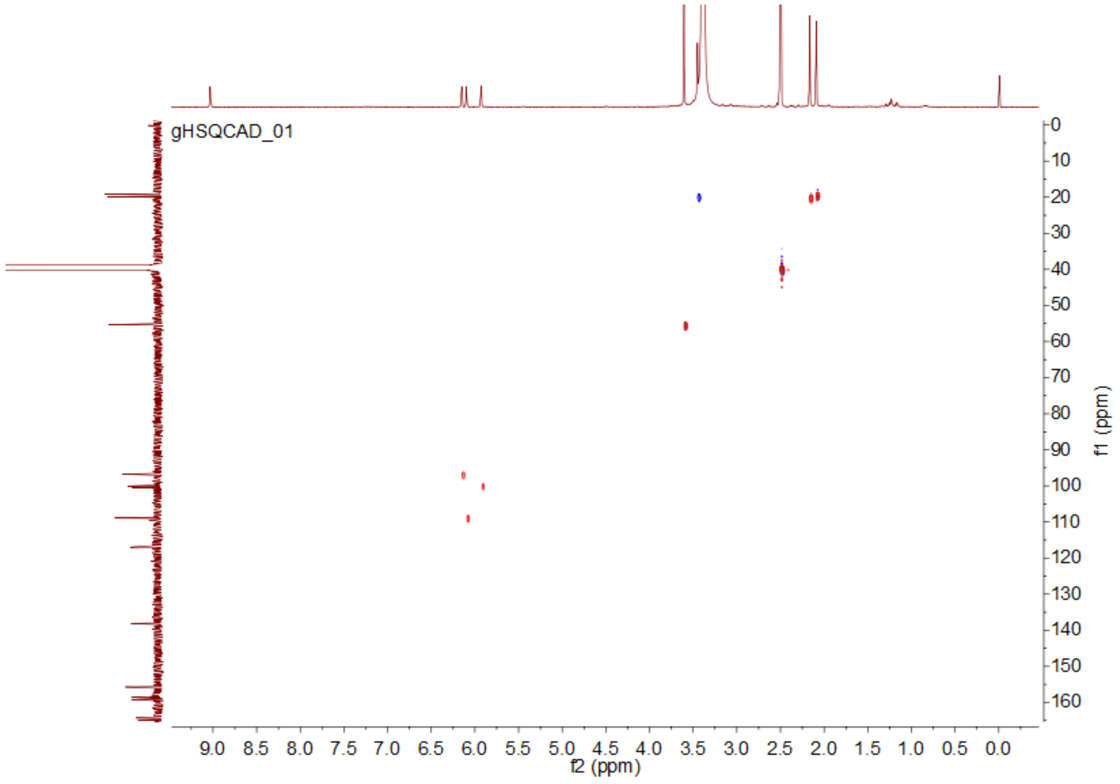

Figure S10. HSQC (DMSO-d_6) spectrum of compound **2**

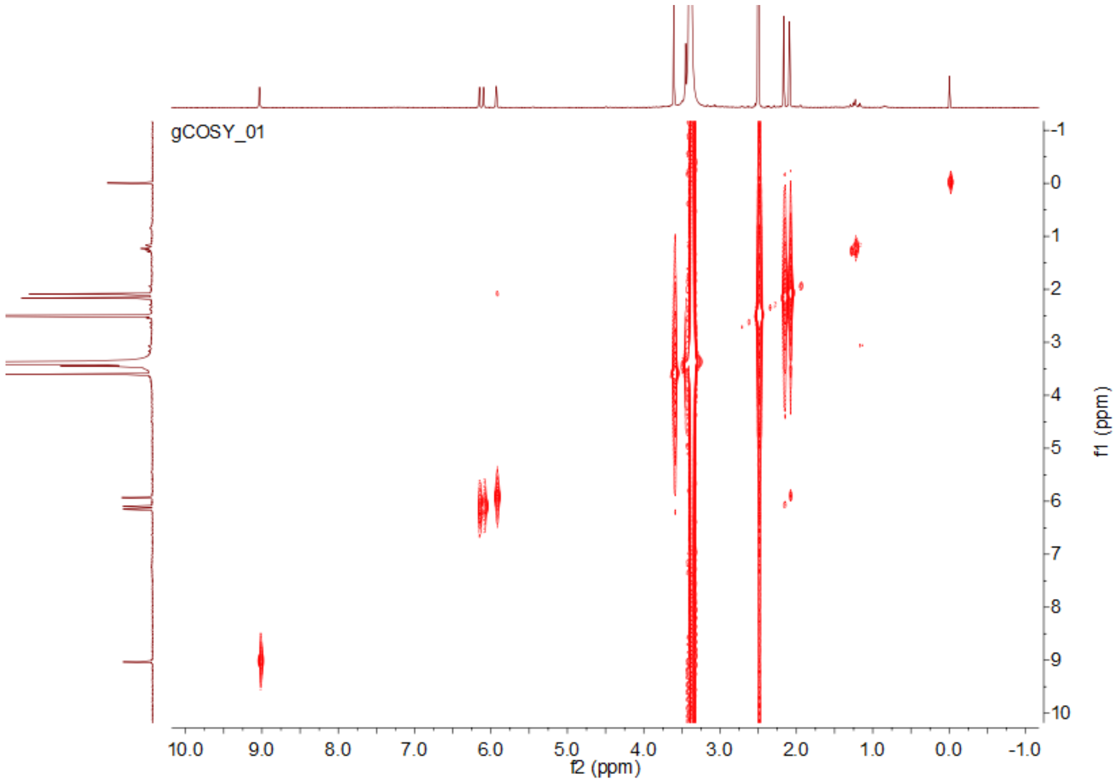

Figure S11. ^{1}H–^{1}H COSY (DMSO-d_6) spectrum of compound **2**

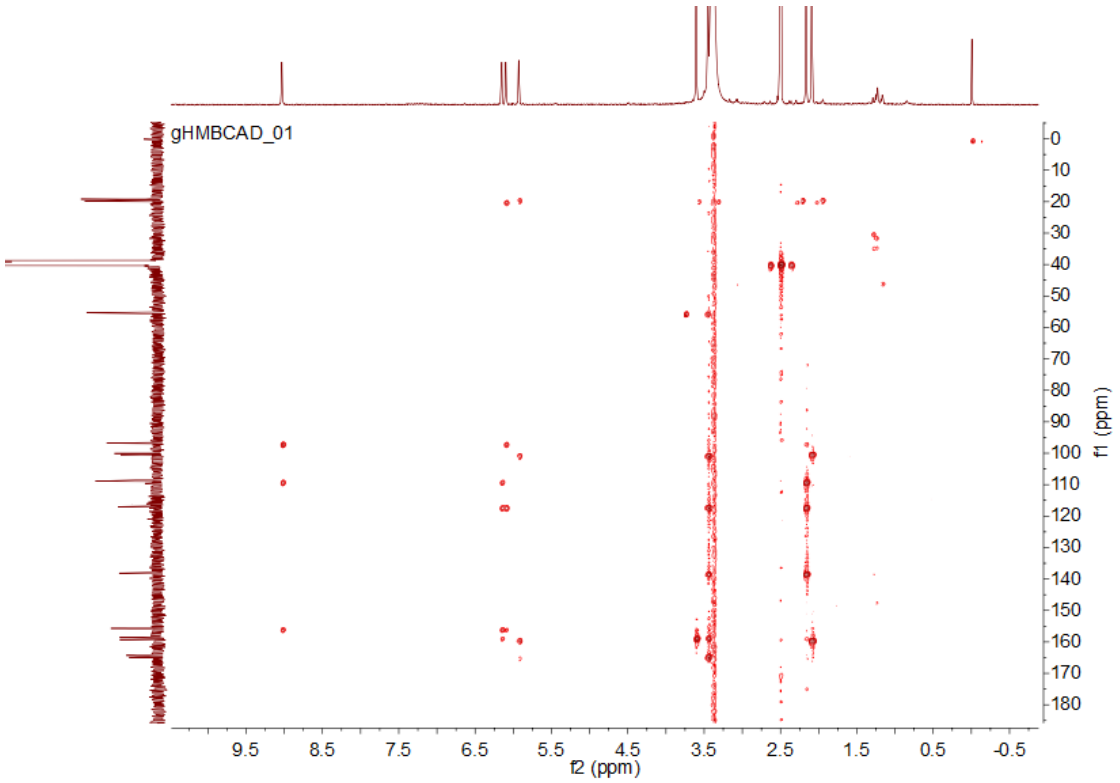

Figure S12. HMBC (DMSO-d_6) spectrum of compound **2**

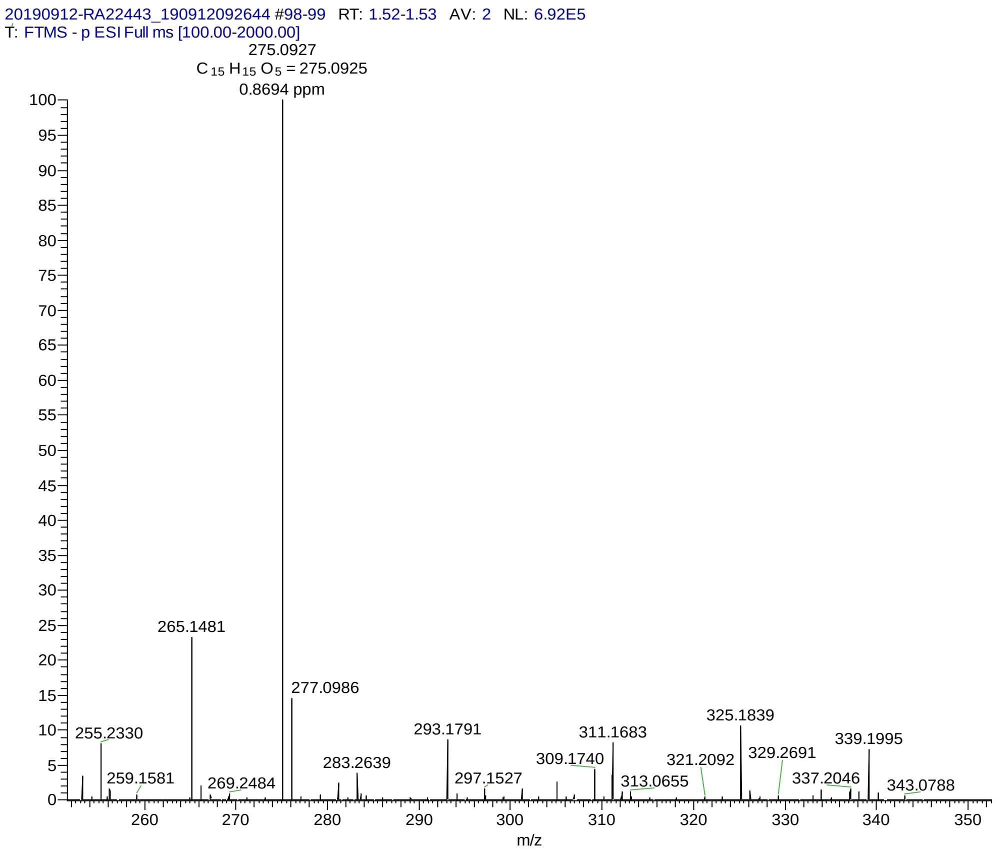

Figure S13. HRESIMS spectrum of compound **2**

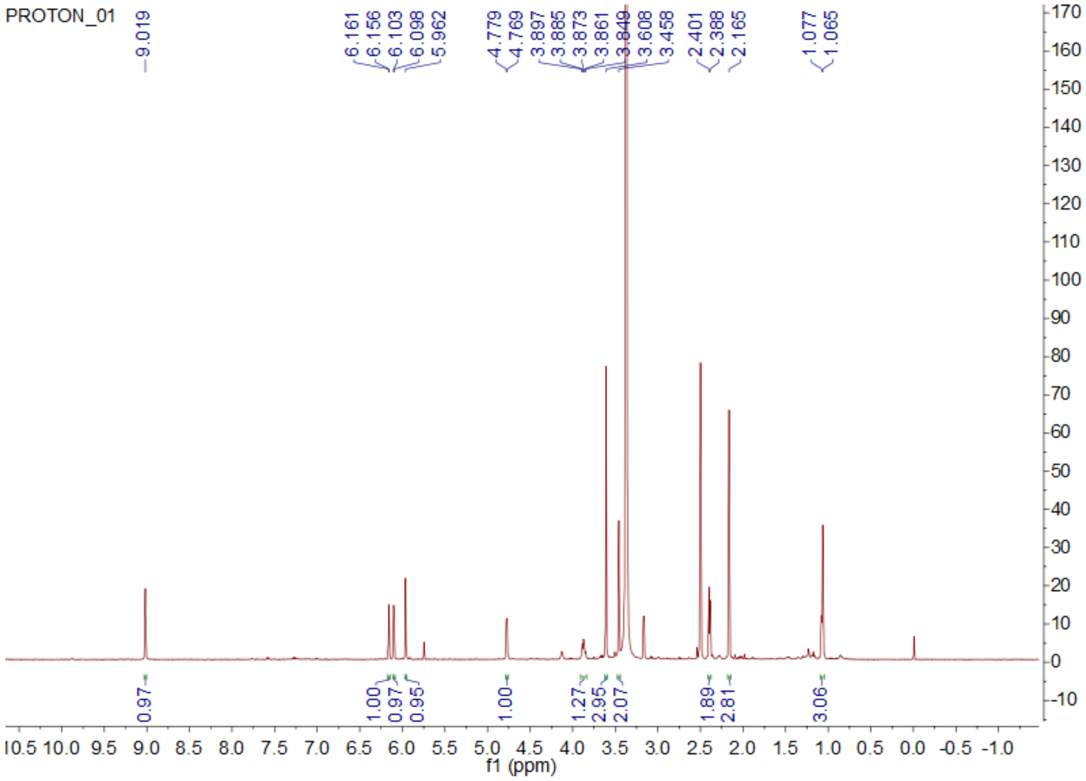

Figure S14. ^{1}H NMR (500 MHz, DMSO-d_6) spectrum of compound **3**

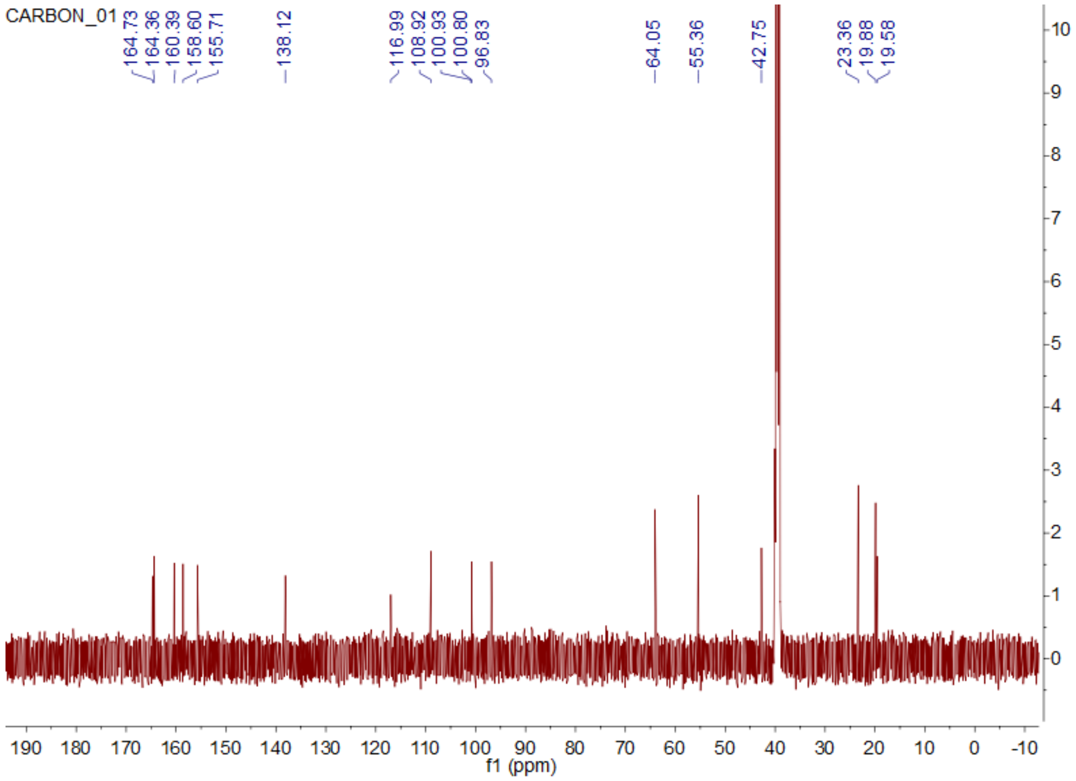

Figure S15. ^{13}C NMR (125 MHz, DMSO-d_6) spectrum of compound **3**

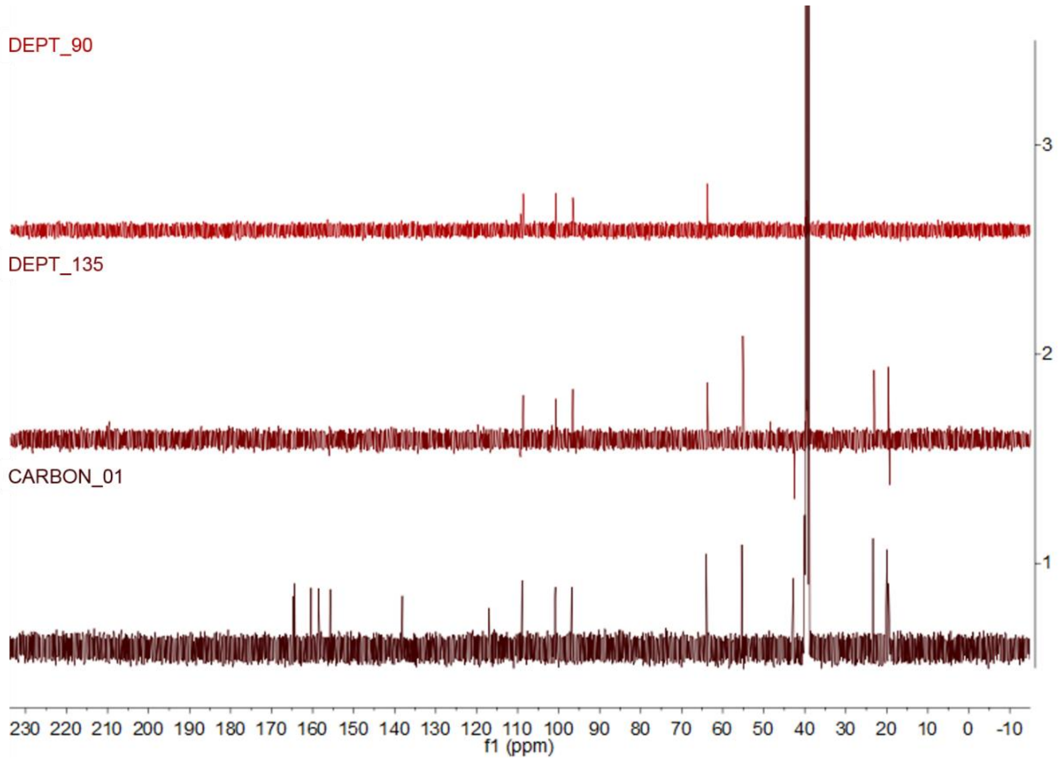

Figure S16. DEPT (125 MHz, DMSO-d_6) spectrum of compound **3**

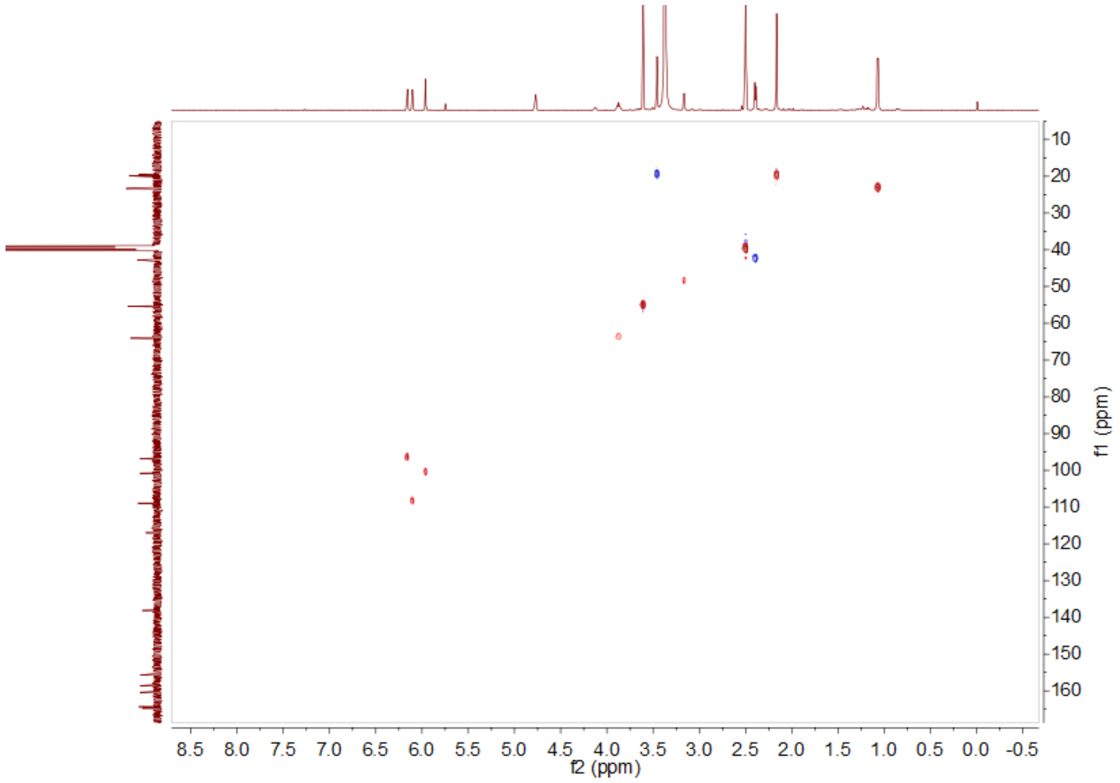

Figure S17. HSQC (DMSO-d_6) spectrum of compound **3**

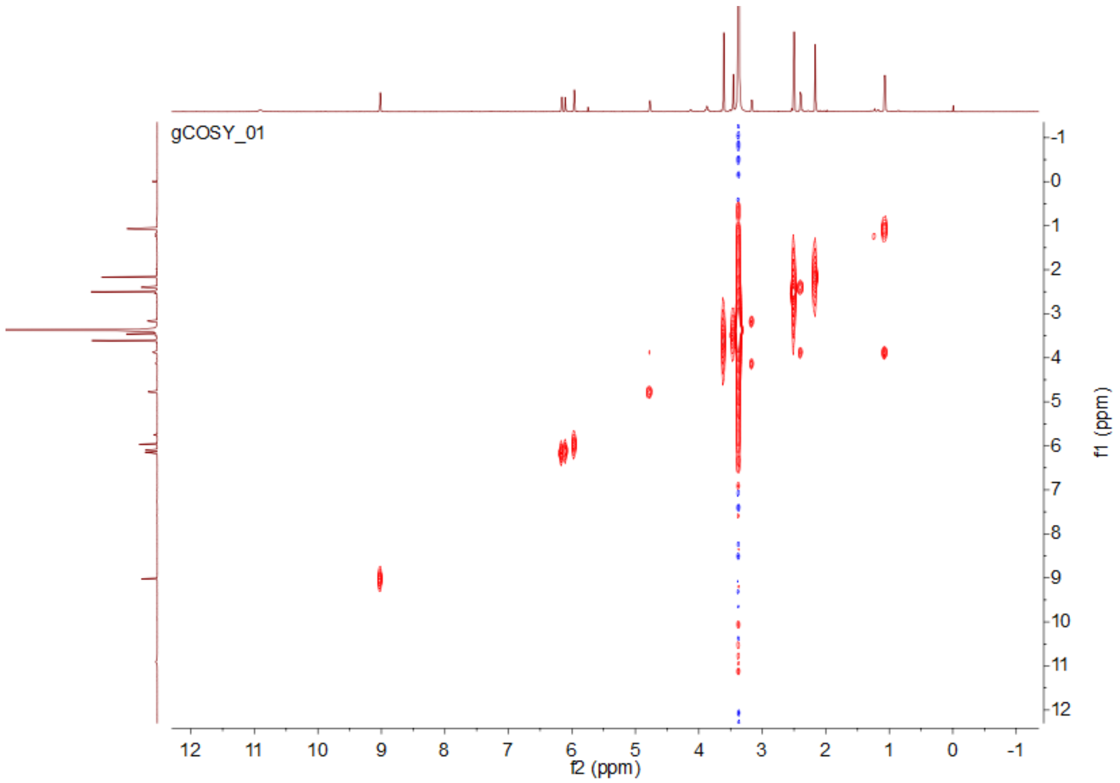

Figure S18. ^{1}H–^{1}H COSY (DMSO-d_6) spectrum of compound **3**

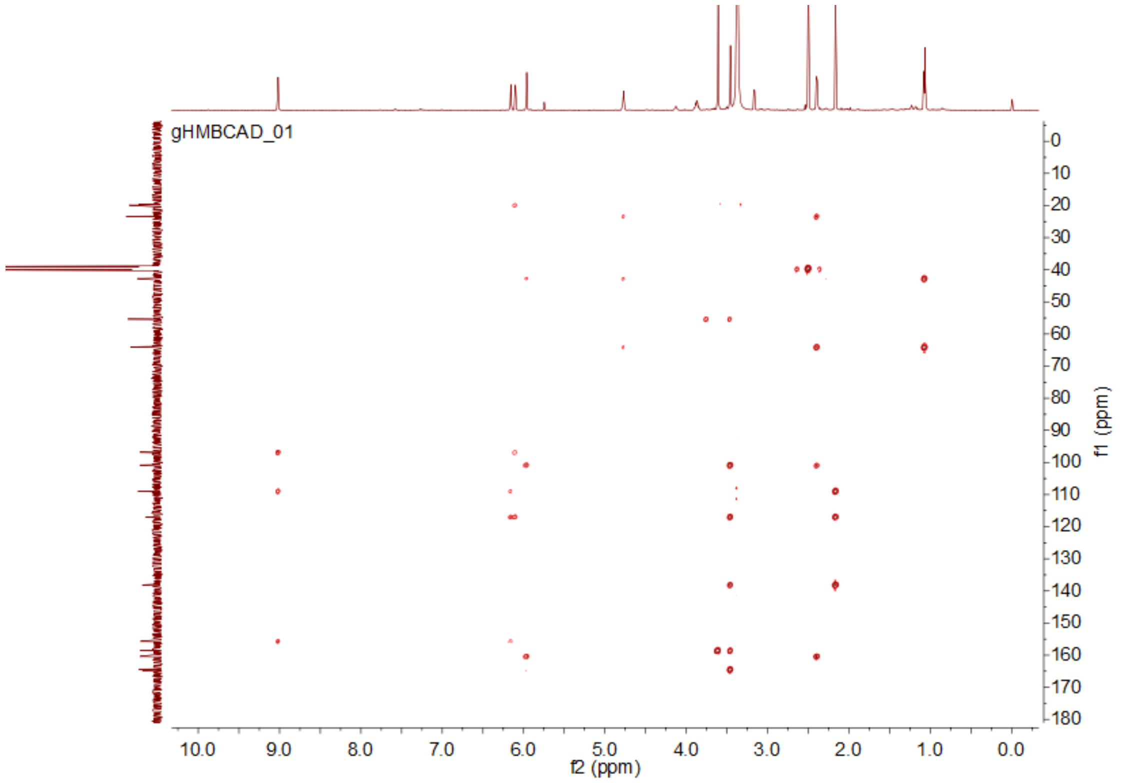

Figure S19. HMBC (DMSO-d_6) spectrum of compound **3**

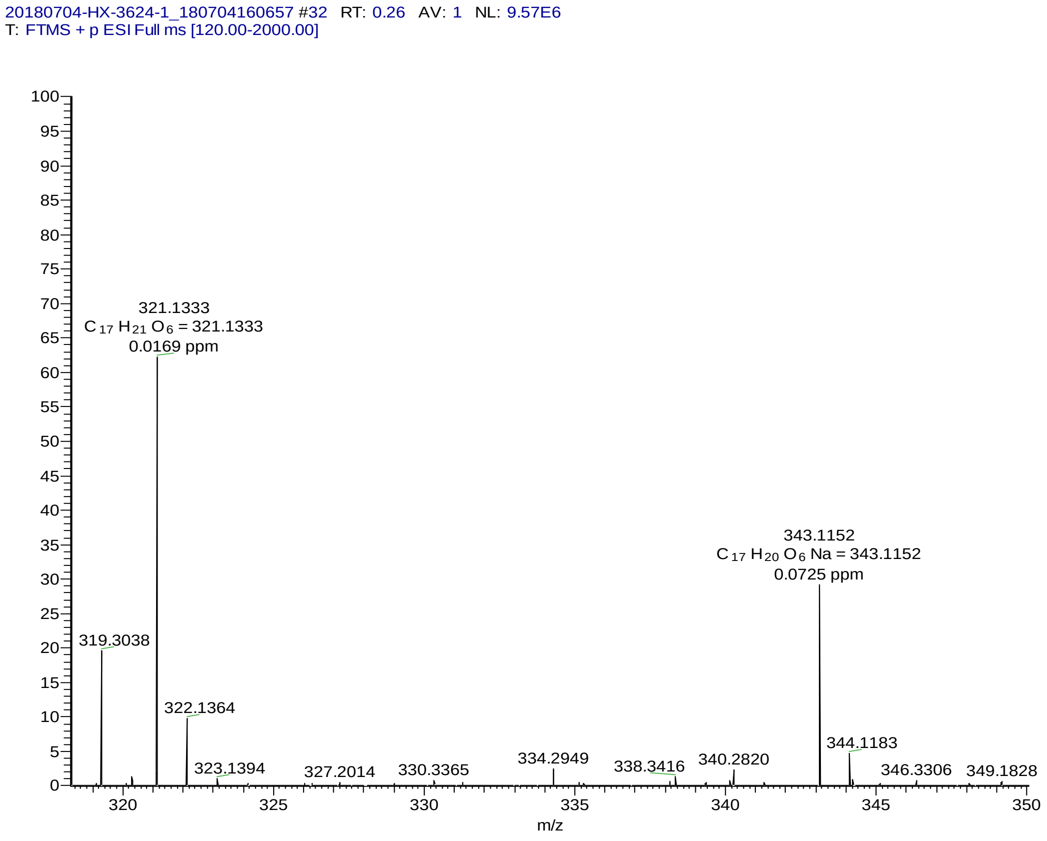

Figure S20. HRESIMS spectrum of compound **3**

Table S1. The ECD calculation results of **(+)-1**, **(−)-1** and **3**

1 For (+)-**1**

Gibbs free energies[a] and equilibrium populations[b] of low-energy conformers of (+)-**1**.

Conformers	In MeOH	
	Δ*G*	*P* (%)
(+)-**1-1**	0.00	49.8
(+)-**1-2**	0.85	11.9
(+)-**1-3**	0.96	9.8
(+)-**1-4**	1.36	5.0

[a]B3LYP/6-31+G(d,p), in kcal/mol. [b]From Δ*G* values at 298.15K.

Cartesian coordinates for the low-energy reoptimized MMFF conformers of (+)-**1** at B3LYP/6-311+G(d,p) level of theory in MeOH.

(+)-**1-1**

(+)-**1-1**		Standard Orientation (Ångstroms)			
No.	Atom	Type	X	Y	Z
1.	6.	0.	-1.991691	2.269669	0.510516
2.	6.	0.	-1.346782	2.561846	1.711541
3.	6.	0.	-0.530331	1.611686	2.328147
4.	6.	0.	-0.371197	0.366865	1.718662
5.	6.	0.	-1.013429	0.045024	0.502222
6.	6.	0.	-1.834165	1.016559	-0.097347
7.	8.	0.	-1.472713	3.769628	2.340665
8.	8.	0.	0.402656	-0.625970	2.243147
9.	6.	0.	1.140441	-0.378911	3.429483
10.	6.	0.	-2.573770	0.732213	-1.386103
11.	6.	0.	-0.828621	-1.327921	-0.103446
12.	6.	0.	0.398734	-1.479300	-1.059242
13.	8.	0.	0.524896	-0.297279	-1.876408
14.	6.	0.	1.705732	0.292131	-1.591939
15.	6.	0.	2.470309	-0.377916	-0.688788
16.	6.	0.	1.749520	-1.568917	-0.309470
17.	6.	0.	0.141764	-2.688491	-1.995948
18.	8.	0.	-0.340696	-2.547972	-3.107299
19.	6.	0.	1.959752	1.562702	-2.322105
20.	8.	0.	2.077952	-2.511649	0.406854
21.	7.	0.	0.412052	-3.880039	-1.404594
22.	1.	0.	-2.628515	3.017216	0.042335
23.	1.	0.	-0.046513	1.866490	3.261731
24.	1.	0.	-2.073738	4.326345	1.828443
25.	1.	0.	0.479081	-0.182939	4.282764

26.	1.	0.	1.714861	-1.287287	3.613119
27.	1.	0.	1.828184	0.466758	3.306319
28.	1.	0.	-1.902088	0.362996	-2.165373
29.	1.	0.	-3.350495	-0.028671	-1.240691
30.	1.	0.	-3.067892	1.633795	-1.757464
31.	1.	0.	-0.697934	-2.079282	0.679438
32.	1.	0.	-1.717702	-1.598517	-0.677819
33.	1.	0.	3.453418	-0.093844	-0.344108
34.	1.	0.	1.895347	1.393443	-3.401844
35.	1.	0.	2.943118	1.965471	-2.075975
36.	1.	0.	1.192799	2.299096	-2.059736
37.	1.	0.	0.305478	-4.710580	-1.967746
38.	1.	0.	0.995177	-3.894452	-0.571940

(+)-**1-2**

(+)-**1-2**		Standard Orientation (Ångstroms)			
No.	Atom	Type	X	Y	Z
1.	6.	0.	-2.033861	2.290785	0.308437
2.	6.	0.	-1.306185	2.765224	1.398389
3.	6.	0.	-0.421191	1.925943	2.080184
4.	6.	0.	-0.275286	0.604594	1.647118
5.	6.	0.	-0.998064	0.102093	0.546379
6.	6.	0.	-1.890150	0.966570	-0.119045
7.	8.	0.	-1.495315	4.069647	1.763230
8.	8.	0.	0.562220	-0.288593	2.249437
9.	6.	0.	1.396764	0.141778	3.311012
10.	6.	0.	-2.722369	0.483944	-1.286621
11.	6.	0.	-0.820560	-1.340788	0.129626
12.	6.	0.	0.332947	-1.606120	-0.890565
13.	8.	0.	0.341930	-0.564303	-1.888294
14.	6.	0.	1.513975	0.098978	-1.800612
15.	6.	0.	2.377335	-0.397173	-0.874046
16.	6.	0.	1.742401	-1.535221	-0.255049
17.	6.	0.	0.056487	-2.956065	-1.602853
18.	8.	0.	-0.511321	-3.009147	-2.680734
19.	6.	0.	1.651258	1.247428	-2.735479
20.	8.	0.	2.168764	-2.342400	0.567701
21.	7.	0.	0.418301	-4.029600	-0.854386
22.	1.	0.	-2.717875	2.965195	-0.195260
23.	1.	0.	0.138106	2.301870	2.930246
24.	1.	0.	-0.949910	4.264120	2.536398

25.	1.	0.	2.048724	0.968356	3.001309
26.	1.	0.	0.811682	0.452582	4.186276
27.	1.	0.	2.010636	-0.720789	3.571683
28.	1.	0.	-2.105457	0.017856	-2.059218
29.	1.	0.	-3.460733	-0.262023	-0.967543
30.	1.	0.	-3.272032	1.313525	-1.738091
31.	1.	0.	-0.608578	-1.969505	0.998066
32.	1.	0.	-1.742532	-1.707233	-0.327918
33.	1.	0.	3.371997	-0.032997	-0.664047
34.	1.	0.	1.502325	0.910904	-3.766557
35.	1.	0.	2.634060	1.711635	-2.643613
36.	1.	0.	0.878700	1.993115	-2.519767
37.	1.	0.	0.307606	-4.941614	-1.271985
38.	1.	0.	1.066201	-3.893568	-0.082808

(+)-**1-3**

(+)-**1-3**		Standard Orientation (Ångstroms)			
No.	Atom	Type	X	Y	Z
1.	6.	0.	-2.044562	2.703325	0.289526
2.	6.	0.	-1.238196	3.051972	1.369407
3.	6.	0.	-0.405645	2.102090	1.962866
4.	6.	0.	-0.373480	0.810183	1.437056
5.	6.	0.	-1.136295	0.445688	0.301413
6.	6.	0.	-2.012563	1.405850	-0.241664
7.	8.	0.	-1.228987	4.307919	1.911386
8.	8.	0.	0.372364	-0.189358	2.004177
9.	6.	0.	1.040335	0.076385	3.226311
10.	6.	0.	-2.987957	1.066816	-1.348667
11.	6.	0.	-1.045930	-0.973151	-0.223313
12.	6.	0.	0.133080	-1.291228	-1.177498
13.	8.	0.	1.409028	-0.973764	-0.559910
14.	6.	0.	2.121997	-2.123254	-0.417338
15.	6.	0.	1.502339	-3.229921	-0.885842
16.	6.	0.	0.200580	-2.836641	-1.405228
17.	6.	0.	0.024312	-0.558108	-2.527003
18.	8.	0.	-0.983821	-0.655716	-3.210187
19.	6.	0.	3.449713	-1.951108	0.231489
20.	8.	0.	-0.699472	-3.507179	-1.870320
21.	7.	0.	1.127079	0.141285	-2.904265
22.	1.	0.	-2.722011	3.438881	-0.138701
23.	1.	0.	0.185110	2.395491	2.820091

24.	1.	0.	-1.852764	4.861006	1.422901
25.	1.	0.	1.826497	0.832882	3.105573
26.	1.	0.	0.343636	0.409343	4.005583
27.	1.	0.	1.495184	-0.867575	3.532136
28.	1.	0.	-3.727213	0.334888	-1.000012
29.	1.	0.	-3.537102	1.958798	-1.661297
30.	1.	0.	-2.501683	0.633206	-2.224366
31.	1.	0.	-0.964397	-1.665535	0.619361
32.	1.	0.	-1.938894	-1.251189	-0.786807
33.	1.	0.	1.884688	-4.239678	-0.862948
34.	1.	0.	4.076736	-1.270418	-0.355169
35.	1.	0.	3.963032	-2.908496	0.330790
36.	1.	0.	3.323532	-1.502689	1.222321
37.	1.	0.	1.059803	0.688498	-3.748822
38.	1.	0.	1.860931	0.326500	-2.240031

(+)-**1-4**

(+)-**1-4**		Standard Orientation (Ångstroms)			
No.	Atom	Type	X	Y	Z
1.	6.	0.	-0.412104	3.014241	-1.294474
2.	6.	0.	0.104437	3.493630	-0.093618
3.	6.	0.	0.837101	2.652528	0.745177
4.	6.	0.	1.018531	1.320167	0.372531
5.	6.	0.	0.466124	0.795213	-0.818880
6.	6.	0.	-0.227541	1.676993	-1.671653
7.	8.	0.	-0.057641	4.788539	0.316608
8.	8.	0.	1.756276	0.444363	1.125614
9.	6.	0.	2.481027	0.948497	2.234859
10.	6.	0.	-0.737257	1.236974	-3.026924
11.	6.	0.	0.711077	-0.651462	-1.195867
12.	6.	0.	-0.227515	-1.721501	-0.583742
13.	8.	0.	0.038205	-1.878661	0.837691
14.	6.	0.	-1.107647	-1.634904	1.527760
15.	6.	0.	-2.176582	-1.346286	0.752129
16.	6.	0.	-1.747248	-1.366643	-0.635848
17.	6.	0.	0.017300	-3.066349	-1.307479
18.	8.	0.	0.009419	-3.124233	-2.525372
19.	6.	0.	-0.972179	-1.727818	3.006355
20.	8.	0.	-2.387357	-1.162582	-1.651270
21.	7.	0.	0.202108	-4.142578	-0.494465
22.	1.	0.	-0.952673	3.686436	-1.957188

23.	1.	0.	1.246296	3.061310	1.659409
24.	1.	0.	-0.576860	5.260753	-0.347511
25.	1.	0.	3.171052	1.748376	1.938658
26.	1.	0.	1.815264	1.328978	3.020616
27.	1.	0.	3.052386	0.105962	2.628301
28.	1.	0.	0.097091	0.988879	-3.695017
29.	1.	0.	-1.377019	0.354279	-2.960558
30.	1.	0.	-1.306163	2.040457	-3.501851
31.	1.	0.	0.618523	-0.786927	-2.274157
32.	1.	0.	1.729770	-0.938927	-0.916262
33.	1.	0.	-3.175832	-1.116086	1.090516
34.	1.	0.	-0.649801	-2.732942	3.300612
35.	1.	0.	-1.918076	-1.500233	3.499599
36.	1.	0.	-0.205421	-1.026593	3.351337
37.	1.	0.	0.443366	-5.016233	-0.937195
38.	1.	0.	0.399562	-4.006616	0.483559

2 For (−)-**1**

Gibbs free energies[a] and equilibrium populations[b] of low-energy conformers of (−)-**1**.

Conformers	In MeOH	
	Δ*G*	*P* (%)
**(−)-1-1**	0	69.47
**(−)-1-2**	0.50	29.94
**(−)-1-3**	3.07	0.39
**(−)-1-4**	3.45	0.21

[a]B3LYP/6-31+G(d,p), in kcal/mol. [b]From Δ*G* values at 298.15K.

Cartesian coordinates for the low-energy reoptimized MMFF conformers of (−)-**1** at B3LYP/6-311+G(d,p) level of theory in MeOH.

(−)-1-1

**(−)-1-1**		Standard Orientation (Ångstroms)			
Center number	Atomic number	Atomic Type	X	Y	Z
1.	6.	0.	0.527477	3.013929	0.133792
2.	6.	0.	0.091010	3.028713	1.457806
3.	6.	0.	0.056075	1.850825	2.206769
4.	6.	0.	0.463907	0.660340	1.604442
5.	6.	0.	0.910859	0.617694	0.264963
6.	6.	0.	0.941431	1.817442	-0.467591
7.	8.	0.	-0.320155	4.172180	2.085339

8.	8.	0.	0.467671	-0.539455	2.252616
9.	6.	0.	-0.023693	-0.613154	3.581485
10.	6.	0.	1.433127	1.851288	-1.897913
11.	6.	0.	1.366842	-0.694231	-0.332373
12.	6.	0.	0.243520	-1.555444	-0.995298
13.	8.	0.	-0.659674	-0.696920	-1.721993
14.	6.	0.	-1.883780	-0.795666	-1.161102
15.	6.	0.	-1.966536	-1.683350	-0.134310
16.	6.	0.	-0.661522	-2.275673	0.032841
17.	6.	0.	0.906154	-2.526099	-2.007401
18.	8.	0.	0.997621	-2.252988	-3.192579
19.	6.	0.	-2.921006	0.078180	-1.771515
20.	8.	0.	-0.279859	-3.180816	0.770928
21.	7.	0.	1.442227	-3.622307	-1.412807
22.	1.	0.	0.553983	3.939270	-0.437696
23.	1.	0.	-0.283634	1.894894	3.233043
24.	1.	0.	-0.230173	4.913451	1.472176
25.	1.	0.	0.028060	-1.667052	3.856150
26.	1.	0.	-1.064436	-0.272297	3.644665
27.	1.	0.	0.590565	-0.022198	4.272391
28.	1.	0.	0.917444	1.115776	-2.520694
29.	1.	0.	1.281313	2.840492	-2.337602
30.	1.	0.	2.505952	1.629869	-1.956286
31.	1.	0.	2.121260	-0.505763	-1.099780
32.	1.	0.	1.822862	-1.327422	0.432918
33.	1.	0.	-2.852994	-1.930440	0.430608
34.	1.	0.	-3.891272	-0.076549	-1.297753
35.	1.	0.	-3.001907	-0.129385	-2.843438
36.	1.	0.	-2.630042	1.128293	-1.662338
37.	1.	0.	1.143657	-3.866825	-0.472241
38.	1.	0.	1.856665	-4.318603	-2.014223

(−)-1-2

**(−)-1-2**		Standard Orientation (Ångstroms)			
Center number	Atom number	Type	X	Y	Z
1.	6.	0.	0.519048	3.033178	0.110080
2.	6.	0.	0.096470	3.038329	1.438243
3.	6.	0.	0.080814	1.855789	2.182415
4.	6.	0.	0.491611	0.667815	1.570252
5.	6.	0.	0.920981	0.636427	0.228173

6.	6.	0.	0.935106	1.844049	-0.498053
7.	8.	0.	-0.296203	4.235401	1.970412
8.	8.	0.	0.512532	-0.534311	2.215567
9.	6.	0.	0.019291	-0.624863	3.541134
10.	6.	0.	1.415697	1.888887	-1.931847
11.	6.	0.	1.375407	-0.670439	-0.382258
12.	6.	0.	0.247887	-1.531878	-1.036836
13.	8.	0.	-0.650612	-0.672413	-1.768257
14.	6.	0.	-1.874562	-0.759108	-1.206227
15.	6.	0.	-1.962421	-1.640656	-0.174169
16.	6.	0.	-0.661341	-2.240467	-0.003990
17.	6.	0.	0.903492	-2.514547	-2.042127
18.	8.	0.	0.990071	-2.254928	-3.230480
19.	6.	0.	-2.905490	0.119587	-1.820134
20.	8.	0.	-0.285738	-3.143640	0.739938
21.	7.	0.	1.439706	-3.605727	-1.437632
22.	1.	0.	0.526270	3.968850	-0.438442
23.	1.	0.	-0.245285	1.869224	3.216930
24.	1.	0.	-0.545986	4.104179	2.894246
25.	1.	0.	0.076906	-1.681064	3.805578
26.	1.	0.	-1.024665	-0.293011	3.606154
27.	1.	0.	0.628296	-0.038457	4.241311
28.	1.	0.	0.897706	1.156403	-2.556264
29.	1.	0.	1.257730	2.881280	-2.361209
30.	1.	0.	2.488738	1.670879	-1.998885
31.	1.	0.	2.119003	-0.474698	-1.158395
32.	1.	0.	1.843857	-1.306410	0.373085
33.	1.	0.	-2.850514	-1.879588	0.391753
34.	1.	0.	-3.877551	-0.028344	-1.347866
35.	1.	0.	-2.985701	-0.089403	-2.891800
36.	1.	0.	-2.608085	1.168095	-1.712527
37.	1.	0.	1.139319	-3.841722	-0.495453
38.	1.	0.	1.846217	-4.310699	-2.034421

(−)-1-3

**(−)-1-3**		Standard Orientation (Ångstroms)			
Center number	Atom number	Type	X	Y	Z
1.	6.	0.	0.220555	3.391321	0.051091
2.	6.	0.	-0.260265	3.286662	1.353318
3.	6.	0.	-0.209062	2.065774	2.027424

4.	6.	0.	0.302533	0.949798	1.364594
5.	6.	0.	0.741062	1.014344	0.019994
6.	6.	0.	0.733725	2.270528	-0.617595
7.	8.	0.	-0.779516	4.350658	2.038532
8.	8.	0.	0.439661	-0.265501	1.982199
9.	6.	0.	0.160052	-0.353806	3.369496
10.	6.	0.	1.332627	2.475978	-1.992704
11.	6.	0.	1.277047	-0.239451	-0.641269
12.	6.	0.	0.231982	-1.206961	-1.252581
13.	8.	0.	-0.752348	-1.610069	-0.263423
14.	6.	0.	-0.648331	-2.951971	-0.066867
15.	6.	0.	0.291716	-3.559116	-0.825689
16.	6.	0.	0.942307	-2.544282	-1.641852
17.	6.	0.	-0.489402	-0.611418	-2.475460
18.	8.	0.	0.146643	-0.172114	-3.421557
19.	6.	0.	-1.585039	-3.502369	0.949475
20.	8.	0.	1.867599	-2.633948	-2.424038
21.	7.	0.	-1.847133	-0.661116	-2.436183
22.	1.	0.	0.216128	4.356529	-0.450622
23.	1.	0.	-0.561596	2.023816	3.049128
24.	1.	0.	-0.745961	5.131542	1.470455
25.	1.	0.	0.414080	-1.373899	3.663344
26.	1.	0.	-0.900619	-0.170320	3.584541
27.	1.	0.	0.767440	0.350345	3.951504
28.	1.	0.	0.915255	1.801613	-2.742608
29.	1.	0.	1.179758	3.505540	-2.326714
30.	1.	0.	2.415003	2.297645	-1.972139
31.	1.	0.	1.949838	-0.002443	-1.467936
32.	1.	0.	1.850567	-0.816050	0.089925
33.	1.	0.	0.543185	-4.609390	-0.820051
34.	1.	0.	-1.441359	-4.576746	1.072138
35.	1.	0.	-2.623367	-3.313726	0.654578
36.	1.	0.	-1.425238	-3.002888	1.910571
37.	1.	0.	-2.321136	-0.877081	-1.574362
38.	1.	0.	-2.347389	-0.216430	-3.190655

(−)-1-4

**(−)-1-4**		Standard Orientation (Ångstroms)			
Center number	Atom number	Type	X	Y	Z
1.	6.	0.	-1.913611	2.637561	-0.559618

2.	6.	0.	-1.981666	2.759826	0.825702
3.	6.	0.	-1.665050	1.676095	1.646130
4.	6.	0.	-1.254401	0.480543	1.055365
5.	6.	0.	-1.132309	0.342818	-0.346875
6.	6.	0.	-1.504631	1.436091	-1.154385
7.	8.	0.	-2.368733	3.914929	1.448005
8.	8.	0.	-0.962264	-0.636925	1.793202
9.	6.	0.	-1.234292	-0.620307	3.184460
10.	6.	0.	-1.533538	1.336717	-2.664175
11.	6.	0.	-0.707735	-0.982622	-0.945157
12.	6.	0.	0.810973	-1.276265	-1.034292
13.	8.	0.	1.361879	-1.515180	0.290571
14.	6.	0.	2.362006	-0.622464	0.517297
15.	6.	0.	2.608069	0.215268	-0.514779
16.	6.	0.	1.674837	-0.097284	-1.583713
17.	6.	0.	1.021520	-2.529687	-1.915737
18.	8.	0.	0.496540	-2.609786	-3.013309
19.	6.	0.	2.996933	-0.730705	1.858329
20.	8.	0.	1.551619	0.412296	-2.682627
21.	7.	0.	1.839706	-3.482794	-1.390808
22.	1.	0.	-2.194939	3.477496	-1.190978
23.	1.	0.	-1.751421	1.795231	2.717909
24.	1.	0.	-2.559012	4.580636	0.774020
25.	1.	0.	-2.287003	-0.388709	3.388983
26.	1.	0.	-0.603258	0.103499	3.716739
27.	1.	0.	-1.008516	-1.624780	3.546640
28.	1.	0.	-2.300542	0.624235	-2.992968
29.	1.	0.	-1.778282	2.305423	-3.107466
30.	1.	0.	-0.578272	1.001543	-3.074029
31.	1.	0.	-1.147376	-1.804905	-0.371477
32.	1.	0.	-1.067223	-1.072095	-1.971014
33.	1.	0.	3.349446	0.999720	-0.546325
34.	1.	0.	3.774371	0.024066	1.983691
35.	1.	0.	3.439160	-1.723883	1.995443
36.	1.	0.	2.238170	-0.602480	2.637109
37.	1.	0.	2.077988	-3.458594	-0.412725
38.	1.	0.	1.930856	-4.344529	-1.907138

3 For 17*S*-**3**

Gibbs free energies[a] and equilibrium populations[b] of low-energy conformers of 17*S*-**3**.

Conformers	In MeOH	
	Δ*G*	*P* (%)
17*S*-**3**-1	0.19	29.48

17*S*-**3**-2	0	40.44
17*S*-**3**-3	0.72	11.89
17*S*-**3**-4	0.83	10.00
17*S*-**3**-5	0.95	8.19

[a]B3LYP/6-31+G(d,p), in kcal/mol. [b]From Δ*G* values at 298.15K.

Cartesian coordinates for the low-energy reoptimized MMFF conformers of 17*S*-**3** at B3LYP/6-311+G(d,p) level of theory in MeOH.

17*S*-**3**-1

17*S*-**3**-1		Standard Orientation (Ångstroms)			
Center number	Atomic number	Atomic Type	X	Y	Z
1.	8.	0.	-2.206597	1.269680	0.983157
2.	6.	0.	-1.425441	1.543059	2.058697
3.	6.	0.	-0.369781	0.754080	2.353670
4.	6.	0.	-0.084466	-0.384730	1.526308
5.	6.	0.	-0.849247	-0.671432	0.411638
6.	6.	0.	-1.973263	0.178806	0.106583
7.	8.	0.	-2.761274	0.078024	-0.813783
8.	6.	0.	-0.633464	-1.884353	-0.489521
9.	6.	0.	0.587612	-1.859597	-1.407118
10.	6.	0.	0.556612	-1.282011	-2.691849
11.	6.	0.	1.702135	-1.304943	-3.498466
12.	6.	0.	2.878299	-1.907741	-3.059049
13.	6.	0.	2.930319	-2.506861	-1.799570
14.	6.	0.	1.789428	-2.474283	-1.002494
15.	8.	0.	1.767244	-3.054048	0.258695
16.	6.	0.	-0.706090	-0.675276	-3.258709
17.	6.	0.	2.907996	-3.784185	0.707908
18.	8.	0.	4.017075	-1.952038	-3.812821
19.	8.	0.	0.959306	-1.112047	1.944096
20.	6.	0.	-1.868663	2.763332	2.805646
21.	6.	0.	-2.023977	4.022936	1.914577
22.	8.	0.	-3.172093	3.937092	1.085976
23.	6.	0.	-2.187894	5.279316	2.762080
24.	1.	0.	0.263471	0.956849	3.207314
25.	1.	0.	-1.535798	-1.977753	-1.096572
26.	1.	0.	-0.582293	-2.785604	0.130991
27.	1.	0.	1.665321	-0.854316	-4.487965
28.	1.	0.	3.853725	-2.971581	-1.481619

29.	1.	0.	-1.434635	-1.457596	-3.506039
30.	1.	0.	-0.490420	-0.126838	-4.179276
31.	1.	0.	-1.208956	-0.004247	-2.561166
32.	1.	0.	3.787959	-3.137877	0.798495
33.	1.	0.	2.642006	-4.177004	1.689875
34.	1.	0.	3.128843	-4.613353	0.028252
35.	1.	0.	3.853716	-1.503100	-4.652725
36.	1.	0.	1.160707	-1.859957	1.337980
37.	1.	0.	-1.147601	2.953612	3.606606
38.	1.	0.	-2.844455	2.573723	3.272218
39.	1.	0.	-1.110685	4.123132	1.302678
40.	1.	0.	-3.126083	3.100625	0.599806
41.	1.	0.	-1.298880	5.464490	3.372829
42.	1.	0.	-3.056516	5.182937	3.421481
43.	1.	0.	-2.351203	6.142076	2.111186

17*S*-**3**-2

17*S*-**3**-2		Standard Orientation (Ångstroms)			
Center number	Atom number	Type	X	Y	Z
1.	8.	0.	-1.137521	1.161110	1.973074
2.	6.	0.	-0.495048	2.279990	1.552106
3.	6.	0.	0.021052	2.342455	0.305519
4.	6.	0.	-0.097161	1.207424	-0.566506
5.	6.	0.	-0.737603	0.052694	-0.158844
6.	6.	0.	-1.328188	0.017246	1.155909
7.	8.	0.	-1.980917	-0.875543	1.660982
8.	6.	0.	-0.958983	-1.164756	-1.052192
9.	6.	0.	0.250843	-2.057445	-1.322775
10.	6.	0.	0.607302	-3.125413	-0.475732
11.	6.	0.	1.718122	-3.922537	-0.782270
12.	6.	0.	2.476306	-3.688428	-1.926841
13.	6.	0.	2.131946	-2.651632	-2.795369
14.	6.	0.	1.029507	-1.861738	-2.480912
15.	8.	0.	0.624243	-0.820379	-3.304671
16.	6.	0.	-0.211519	-3.477137	0.744818
17.	6.	0.	1.302948	-0.609778	-4.542163
18.	8.	0.	3.567202	-4.439795	-2.262151
19.	8.	0.	0.446776	1.390983	-1.776194
20.	6.	0.	-0.462876	3.358728	2.590628
21.	6.	0.	-1.857988	3.716637	3.165840

22.	8.	0.	-2.342464	2.700687	4.028884
23.	6.	0.	-1.801670	4.999757	3.986687
24.	1.	0.	0.529712	3.229065	-0.049420
25.	1.	0.	-1.365660	-0.832005	-2.013377
26.	1.	0.	-1.741158	-1.756929	-0.573636
27.	1.	0.	1.983363	-4.743326	-0.119379
28.	1.	0.	2.733010	-2.492309	-3.680176
29.	1.	0.	-0.440363	-2.612650	1.369460
30.	1.	0.	0.306003	-4.222513	1.354115
31.	1.	0.	-1.178603	-3.905539	0.453213
32.	1.	0.	1.236288	-1.499402	-5.176505
33.	1.	0.	0.790026	0.222857	-5.024830
34.	1.	0.	2.355037	-0.348938	-4.383099
35.	1.	0.	3.701508	-5.115668	-1.584787
36.	1.	0.	0.408057	0.578043	-2.328714
37.	1.	0.	0.002001	4.244933	2.147531
38.	1.	0.	0.163536	3.038771	3.433871
39.	1.	0.	-2.548443	3.866836	2.317828
40.	1.	0.	-2.318128	1.862462	3.544078
41.	1.	0.	-1.516529	5.855021	3.366278
42.	1.	0.	-1.079069	4.898704	4.803000
43.	1.	0.	-2.782896	5.197454	4.425721

17*S*-**3**-3

**17*S*-3**-3		Standard Orientation (Ångstroms)			
Center number	Atom number	Type	X	Y	Z
1.	8.	0.	-0.853112	2.717816	0.221278
2.	6.	0.	-0.345587	2.594654	1.468453
3.	6.	0.	0.627015	1.692856	1.737792
4.	6.	0.	1.134963	0.867738	0.676492
5.	6.	0.	0.628713	0.964172	-0.604235
6.	6.	0.	-0.399437	1.944432	-0.872503
7.	8.	0.	-0.924369	2.190691	-1.941351
8.	6.	0.	1.149677	0.162534	-1.793724
9.	6.	0.	0.778392	-1.318256	-1.848165
10.	6.	0.	-0.411574	-1.777485	-2.446850
11.	6.	0.	-0.694488	-3.149438	-2.482506
12.	6.	0.	0.193515	-4.080064	-1.948824
13.	6.	0.	1.390936	-3.654679	-1.371661
14.	6.	0.	1.661601	-2.289487	-1.335163

15.	8.	0.	2.836915	-1.796934	-0.784673
16.	6.	0.	-1.383545	-0.829754	-3.111596
17.	6.	0.	3.827336	-2.716090	-0.326059
18.	8.	0.	-0.043277	-5.425775	-1.961945
19.	8.	0.	2.117307	0.038250	1.052679
20.	6.	0.	-1.025732	3.483796	2.462575
21.	6.	0.	-2.364674	2.884626	2.961612
22.	8.	0.	-2.141281	1.676361	3.679864
23.	6.	0.	-3.084758	3.833780	3.909404
24.	1.	0.	1.045640	1.597775	2.731313
25.	1.	0.	0.768309	0.660101	-2.687076
26.	1.	0.	2.241073	0.248281	-1.831608
27.	1.	0.	-1.617519	-3.490455	-2.946452
28.	1.	0.	2.066703	-4.396737	-0.968612
29.	1.	0.	-1.610652	0.047908	-2.505023
30.	1.	0.	-2.318494	-1.342808	-3.351393
31.	1.	0.	-0.968541	-0.448369	-4.053169
32.	1.	0.	3.453668	-3.327703	0.502512
33.	1.	0.	4.159973	-3.365455	-1.142117
34.	1.	0.	4.662456	-2.105745	0.019437
35.	1.	0.	-0.898159	-5.588533	-2.381874
36.	1.	0.	2.406707	-0.550983	0.320337
37.	1.	0.	-1.213197	4.456432	1.995109
38.	1.	0.	-0.372126	3.632930	3.327626
39.	1.	0.	-3.001424	2.696850	2.082371
40.	1.	0.	-1.678961	1.062315	3.091902
41.	1.	0.	-3.331792	4.774047	3.407517
42.	1.	0.	-2.456980	4.052739	4.779237
43.	1.	0.	-4.010221	3.373665	4.265071

17*S*-**3**-4

17*S*-**3**-4		Standard Orientation (Ångstroms)			
Center number	Atom number	Type	X	Y	Z
1.	8.	0.	-0.043867	2.578582	-0.579766
2.	6.	0.	-0.100782	2.685732	0.767017
3.	6.	0.	-0.400998	1.614631	1.538034
4.	6.	0.	-0.633072	0.341234	0.913659
5.	6.	0.	-0.556032	0.196438	-0.456902
6.	6.	0.	-0.274612	1.362398	-1.263821
7.	8.	0.	-0.216367	1.424511	-2.476806

8.	6.	0.	-0.842241	-1.106898	-1.197254
9.	6.	0.	0.216463	-2.204281	-1.108345
10.	6.	0.	1.298830	-2.285048	-2.006731
11.	6.	0.	2.231242	-3.324463	-1.889424
12.	6.	0.	2.099736	-4.299458	-0.903749
13.	6.	0.	1.024052	-4.257106	-0.015508
14.	6.	0.	0.105739	-3.217513	-0.135034
15.	8.	0.	-0.991657	-3.117262	0.709184
16.	6.	0.	1.460582	-1.304144	-3.145267
17.	6.	0.	-1.228102	-4.149188	1.665962
18.	8.	0.	2.985934	-5.328784	-0.753623
19.	8.	0.	-0.931725	-0.642055	1.773129
20.	6.	0.	0.117895	4.086637	1.247701
21.	6.	0.	-1.162426	4.951391	1.127823
22.	8.	0.	-2.190118	4.453032	1.977150
23.	6.	0.	-0.906431	6.392736	1.545872
24.	1.	0.	-0.444150	1.693211	2.616595
25.	1.	0.	-0.990167	-0.836195	-2.244150
26.	1.	0.	-1.795076	-1.515673	-0.843649
27.	1.	0.	3.063012	-3.374602	-2.588832
28.	1.	0.	0.941144	-5.028242	0.738352
29.	1.	0.	0.699913	-1.478298	-3.916751
30.	1.	0.	2.438738	-1.421783	-3.618758
31.	1.	0.	1.343598	-0.264681	-2.836102
32.	1.	0.	-1.355565	-5.116146	1.168934
33.	1.	0.	-0.412774	-4.214284	2.394830
34.	1.	0.	-2.152194	-3.875640	2.176409
35.	1.	0.	3.680331	-5.242864	-1.420014
36.	1.	0.	-1.027578	-1.511852	1.323862
37.	1.	0.	0.920945	4.540836	0.657328
38.	1.	0.	0.424529	4.069264	2.298102
39.	1.	0.	-1.489101	4.930372	0.075985
40.	1.	0.	-2.362641	3.534329	1.726947
41.	1.	0.	-0.154202	6.858637	0.902363
42.	1.	0.	-0.556968	6.432245	2.582623
43.	1.	0.	-1.831836	6.970242	1.476624

17*S*-**3**-5

17*S*-**3**-5		Standard Orientation (Ångstroms)			
Center number	Atom number	Type	X	Y	Z

1.	8.	0.	-1.708292	1.525678	1.807572
2.	6.	0.	-1.047273	2.628742	1.372611
3.	6.	0.	-0.461677	2.638381	0.155751
4.	6.	0.	-0.551532	1.475413	-0.682170
5.	6.	0.	-1.196300	0.330759	-0.253748
6.	6.	0.	-1.818362	0.332963	1.047374
7.	8.	0.	-2.437338	-0.566124	1.582936
8.	6.	0.	-1.366627	-0.927000	-1.101514
9.	6.	0.	-0.123001	-1.784868	-1.327620
10.	6.	0.	0.269181	-2.798819	-0.431325
11.	6.	0.	1.410012	-3.567378	-0.697812
12.	6.	0.	2.163130	-3.359120	-1.850766
13.	6.	0.	1.784640	-2.376585	-2.766890
14.	6.	0.	0.653210	-1.613705	-2.491292
15.	8.	0.	0.215254	-0.623571	-3.360096
16.	6.	0.	-0.542020	-3.124624	0.801388
17.	6.	0.	0.885311	-0.448483	-4.607855
18.	8.	0.	3.281722	-4.084944	-2.148916
19.	8.	0.	0.031069	1.621507	-1.878994
20.	6.	0.	-1.068546	3.748882	2.367687
21.	6.	0.	-0.601503	3.357944	3.795504
22.	8.	0.	-1.576004	2.576534	4.469563
23.	6.	0.	0.778307	2.694050	3.818997
24.	1.	0.	0.068935	3.508918	-0.206796
25.	1.	0.	-1.776737	-0.647040	-2.077851
26.	1.	0.	-2.131112	-1.528252	-0.606010
27.	1.	0.	1.702672	-4.346256	0.003035
28.	1.	0.	2.383103	-2.235857	-3.656647
29.	1.	0.	-0.817281	-2.240518	1.377888
30.	1.	0.	0.004712	-3.811868	1.452152
31.	1.	0.	-1.485403	-3.613143	0.526877
32.	1.	0.	0.849396	-1.369159	-5.198851
33.	1.	0.	0.343060	0.341685	-5.128338
34.	1.	0.	1.927757	-0.143520	-4.464291
35.	1.	0.	3.438164	-4.723640	-1.440916
36.	1.	0.	-0.004682	0.798065	-2.415531
37.	1.	0.	-2.094585	4.127321	2.457663
38.	1.	0.	-0.447854	4.563311	1.980848
39.	1.	0.	-0.548337	4.292422	4.367002
40.	1.	0.	-1.763469	1.803462	3.916326
41.	1.	0.	1.540972	3.336476	3.364751
42.	1.	0.	0.769545	1.743296	3.275517
43.	1.	0.	1.067784	2.488667	4.852784

4 For 17*R*-**3**

Gibbs free energies[a] and equilibrium populations[b] of low-energy conformers of 17*R*-**3**.

Conformers	In MeOH	
	Δ*G*	*P* (%)
17*R*-**3**-1	0.19	29.53
17*R*-**3**-2	0	40.46
17*R*-**3**-3	0.73	11.86
17*R*-**3**-4	0.83	9.97
17*R*-**3**-5	0.95	8.17

[a]B3LYP/6-31G(d,p), in kcal/mol. [b]From Δ*G* values at 298.15K.

Gibbs free energies[a] and equilibrium populations[b] of low-energy conformers of 17*R*-**3** in MeOH.

17*R*-**3**-1

17*R*-**3**-1		Standard Orientation (Ångstroms)			
Center number	Atomic number	Atomic Type	X	Y	Z
1.	8.	0.	-1.247813	2.298546	0.779738
2.	6.	0.	-0.379228	2.916237	-0.060351
3.	6.	0.	0.167695	2.240758	-1.094047
4.	6.	0.	-0.184308	0.864444	-1.305419
5.	6.	0.	-1.047120	0.205119	-0.450771
6.	6.	0.	-1.624113	0.936846	0.649693
7.	8.	0.	-2.406213	0.534597	1.489167
8.	6.	0.	-1.499735	-1.240986	-0.634296
9.	6.	0.	-0.471864	-2.332879	-0.342979
10.	6.	0.	-0.281472	-2.867707	0.946509
11.	6.	0.	0.662785	-3.882364	1.152055
12.	6.	0.	1.413309	-4.390361	0.094482
13.	6.	0.	1.229403	-3.894173	-1.196996
14.	6.	0.	0.293380	-2.882160	-1.391134
15.	8.	0.	0.054408	-2.344803	-2.648666
16.	6.	0.	-1.111890	-2.413248	2.124538
17.	6.	0.	0.712224	-2.909613	-3.781997
18.	8.	0.	2.343890	-5.378524	0.250992
19.	8.	0.	0.388715	0.316560	-2.384532
20.	6.	0.	-0.128711	4.346101	0.308689
21.	6.	0.	0.308354	4.546600	1.783009
22.	8.	0.	-0.774805	4.355019	2.678586
23.	6.	0.	0.824735	5.961779	2.015887
24.	1.	0.	0.861592	2.717398	-1.773863

25.	1.	0.	-1.865115	-1.373520	-1.658423
26.	1.	0.	-2.361785	-1.376441	0.021430
27.	1.	0.	0.800927	-4.285917	2.152852
28.	1.	0.	1.821395	-4.306713	-2.002659
29.	1.	0.	-1.184756	-1.327798	2.203334
30.	1.	0.	-0.702549	-2.806007	3.058845
31.	1.	0.	-2.141933	-2.781187	2.037457
32.	1.	0.	0.338508	-2.360666	-4.647053
33.	1.	0.	1.799323	-2.791198	-3.715294
34.	1.	0.	0.463259	-3.970586	-3.884845
35.	1.	0.	2.378673	-5.630421	1.183197
36.	1.	0.	0.166691	-0.636536	-2.482774
37.	1.	0.	-1.046976	4.929702	0.160364
38.	1.	0.	0.632715	4.744826	-0.368740
39.	1.	0.	1.121049	3.831316	1.998566
40.	1.	0.	-1.169509	3.491416	2.487420
41.	1.	0.	0.055321	6.695507	1.754636
42.	1.	0.	1.720221	6.160384	1.418973
43.	1.	0.	1.072322	6.092654	3.072422

17*R*-**3**-2

17*R*-**3**-2		Standard Orientation (Ångstroms)			
Center number	Atom number	Type	X	Y	Z
1.	8.	0.	0.502403	2.505475	-0.075758
2.	6.	0.	0.883874	2.363426	1.218900
3.	6.	0.	0.515022	1.271597	1.923244
4.	6.	0.	-0.268242	0.250300	1.285958
5.	6.	0.	-0.661239	0.365867	-0.033868
6.	6.	0.	-0.303986	1.559448	-0.759394
7.	8.	0.	-0.611456	1.867188	-1.894711
8.	6.	0.	-1.548361	-0.645256	-0.754927
9.	6.	0.	-0.900615	-1.963886	-1.173788
10.	6.	0.	-0.239627	-2.123418	-2.407790
11.	6.	0.	0.322242	-3.361341	-2.747122
12.	6.	0.	0.226699	-4.453242	-1.887869
13.	6.	0.	-0.442583	-4.329453	-0.669426
14.	6.	0.	-0.993940	-3.094763	-0.338147
15.	8.	0.	-1.681890	-2.903102	0.852333
16.	6.	0.	-0.159276	-1.000143	-3.415607
17.	6.	0.	-1.899563	-4.018406	1.715516

18.	8.	0.	0.761340	-5.676641	-2.178547
19.	8.	0.	-0.576643	-0.778242	2.085913
20.	6.	0.	1.696913	3.518616	1.716630
21.	6.	0.	1.025029	4.898940	1.496387
22.	8.	0.	1.053438	5.280853	0.130754
23.	6.	0.	1.748512	5.992806	2.273210
24.	1.	0.	0.803648	1.145486	2.958415
25.	1.	0.	-1.932582	-0.137400	-1.641458
26.	1.	0.	-2.416181	-0.870333	-0.125463
27.	1.	0.	0.830966	-3.470957	-3.702452
28.	1.	0.	-0.507843	-5.192631	-0.021192
29.	1.	0.	-1.143882	-0.804893	-3.858581
30.	1.	0.	0.520044	-1.261968	-4.230848
31.	1.	0.	0.167305	-0.055897	-2.977849
32.	1.	0.	-2.485828	-3.637023	2.552285
33.	1.	0.	-2.462792	-4.802787	1.199972
34.	1.	0.	-0.954027	-4.427999	2.087570
35.	1.	0.	1.196900	-5.630855	-3.039856
36.	1.	0.	-1.057398	-1.490538	1.607262
37.	1.	0.	2.662667	3.540616	1.194454
38.	1.	0.	1.897082	3.361270	2.781045
39.	1.	0.	-0.015416	4.833750	1.859244
40.	1.	0.	0.660717	4.562520	-0.386951
41.	1.	0.	2.800701	6.040520	1.974286
42.	1.	0.	1.695335	5.814430	3.351656
43.	1.	0.	1.290533	6.961068	2.055569

17*R*-**3**-3

17*R*-**3**-3		Standard Orientation (Ångstroms)			
Center number	Atom number	Type	X	Y	Z
1.	8.	0.	0.022365	2.857073	0.010002
2.	6.	0.	0.296917	2.678278	1.321815
3.	6.	0.	-0.087482	1.551625	1.965730
4.	6.	0.	-0.810963	0.540972	1.244380
5.	6.	0.	-1.096305	0.691554	-0.097915
6.	6.	0.	-0.689134	1.909408	-0.762005
7.	8.	0.	-0.892354	2.223220	-1.919095
8.	6.	0.	-1.903586	-0.309572	-0.919470
9.	6.	0.	-1.208317	-1.615642	-1.299390
10.	6.	0.	-0.429697	-1.745541	-2.466528

11.	6.	0.	0.172984	-2.972539	-2.774461
12.	6.	0.	0.002265	-4.083164	-1.951785
13.	6.	0.	-0.783831	-3.989233	-0.802386
14.	6.	0.	-1.374272	-2.765018	-0.500697
15.	8.	0.	-2.175696	-2.602442	0.621043
16.	6.	0.	-0.264373	-0.603149	-3.442445
17.	6.	0.	-2.471123	-3.737543	1.433675
18.	8.	0.	0.571997	-5.297127	-2.214239
19.	8.	0.	-1.168445	-0.512095	1.991255
20.	6.	0.	1.105756	3.795873	1.903374
21.	6.	0.	2.614602	3.653738	1.580902
22.	8.	0.	3.154350	2.486974	2.191660
23.	6.	0.	3.414315	4.837025	2.108177
24.	1.	0.	0.118830	1.407871	3.018413
25.	1.	0.	-2.203297	0.213922	-1.828986
26.	1.	0.	-2.826395	-0.551520	-0.381122
27.	1.	0.	0.772813	-3.058783	-3.677948
28.	1.	0.	-0.905641	-4.866904	-0.182211
29.	1.	0.	-1.196880	-0.428025	-3.993812
30.	1.	0.	0.510803	-0.834077	-4.177722
31.	1.	0.	-0.018198	0.342943	-2.958600
32.	1.	0.	-1.564557	-4.152221	1.887999
33.	1.	0.	-3.138948	-3.377074	2.216864
34.	1.	0.	-2.976519	-4.511840	0.847746
35.	1.	0.	1.088709	-5.230405	-3.028001
36.	1.	0.	-1.612790	-1.210452	1.460097
37.	1.	0.	0.986145	3.806739	2.991184
38.	1.	0.	0.734923	4.745154	1.502083
39.	1.	0.	2.724355	3.600076	0.485982
40.	1.	0.	2.652908	1.724273	1.870201
41.	1.	0.	3.297956	4.922384	3.193456
42.	1.	0.	3.082832	5.770544	1.643765
43.	1.	0.	4.475976	4.695721	1.890015

17*R*-**3**-4

17*R*-**3**-4		Standard Orientation (Ångstroms)			
Center number	Atom number	Type	X	Y	Z
1.	8.	0.	-1.596949	1.915330	0.876462
2.	6.	0.	-0.787570	2.679418	0.108594
3.	6.	0.	0.038889	2.117740	-0.804277

4.	6.	0.	0.074175	0.687050	-0.935142
5.	6.	0.	-0.721748	-0.119218	-0.146613
6.	6.	0.	-1.626742	0.503805	0.792854
7.	8.	0.	-2.424600	-0.047596	1.526314
8.	6.	0.	-0.781539	-1.639587	-0.263349
9.	6.	0.	0.407977	-2.426480	0.283332
10.	6.	0.	0.489298	-2.836047	1.628984
11.	6.	0.	1.596367	-3.571945	2.072145
12.	6.	0.	2.620639	-3.925515	1.197317
13.	6.	0.	2.554211	-3.553338	-0.146159
14.	6.	0.	1.453922	-2.816942	-0.576765
15.	8.	0.	1.318830	-2.419541	-1.899965
16.	6.	0.	-0.621128	-2.551292	2.614115
17.	6.	0.	2.288197	-2.847317	-2.855756
18.	8.	0.	3.717512	-4.641012	1.587618
19.	8.	0.	0.919745	0.245401	-1.875726
20.	6.	0.	-0.984665	4.145748	0.337596
21.	6.	0.	-2.218629	4.694509	-0.422393
22.	8.	0.	-2.033695	4.588245	-1.829653
23.	6.	0.	-2.453852	6.167835	-0.119485
24.	1.	0.	0.691822	2.723714	-1.418933
25.	1.	0.	-0.922046	-1.910879	-1.315343
26.	1.	0.	-1.687240	-1.949793	0.260700
27.	1.	0.	1.647398	-3.880992	3.113976
28.	1.	0.	3.359730	-3.843764	-0.806931
29.	1.	0.	-0.964063	-1.516208	2.586870
30.	1.	0.	-0.302764	-2.789125	3.632399
31.	1.	0.	-1.501433	-3.168140	2.393442
32.	1.	0.	2.328713	-3.940189	-2.903505
33.	1.	0.	1.954579	-2.451353	-3.815570
34.	1.	0.	3.282786	-2.451656	-2.622327
35.	1.	0.	3.646624	-4.830969	2.532360
36.	1.	0.	0.965871	-0.736821	-1.902273
37.	1.	0.	-0.099513	4.691460	-0.003538
38.	1.	0.	-1.110367	4.321315	1.411422
39.	1.	0.	-3.098897	4.113015	-0.105351
40.	1.	0.	-1.878639	3.656360	-2.039239
41.	1.	0.	-1.578638	6.759960	-0.405717
42.	1.	0.	-2.650209	6.321358	0.945827
43.	1.	0.	-3.312355	6.532457	-0.689316

17*R*-**3**-5

17*R*-**3**-5		Standard Orientation (Ångstroms)			
Center number	Atom number	Type	X	Y	Z
1.	8.	0.	1.019812	2.728440	0.170614
2.	6.	0.	0.637138	2.704792	1.472877
3.	6.	0.	-0.354545	1.882664	1.878383
4.	6.	0.	-1.010967	1.038834	0.919351
5.	6.	0.	-0.624062	1.024649	-0.407151
6.	6.	0.	0.435661	1.907597	-0.828188
7.	8.	0.	0.896848	2.044682	-1.944801
8.	6.	0.	-1.299088	0.186339	-1.489395
9.	6.	0.	-1.042809	-1.319384	-1.461307
10.	6.	0.	0.061388	-1.910481	-2.106750
11.	6.	0.	0.237843	-3.299780	-2.063058
12.	6.	0.	-0.674089	-4.119185	-1.402142
13.	6.	0.	-1.788830	-3.562381	-0.773462
14.	6.	0.	-1.953183	-2.180647	-0.816806
15.	8.	0.	-3.040807	-1.559233	-0.218549
16.	6.	0.	1.049274	-1.087635	-2.900967
17.	6.	0.	-4.061454	-2.362954	0.372094
18.	8.	0.	-0.539982	-5.477289	-1.334831
19.	8.	0.	-2.003736	0.301771	1.433447
20.	6.	0.	1.417513	3.658718	2.325160
21.	6.	0.	2.959340	3.524752	2.204956
22.	8.	0.	3.435510	4.043571	0.972596
23.	6.	0.	3.463388	2.100918	2.457570
24.	1.	0.	-0.672351	1.851165	2.912226
25.	1.	0.	-0.958892	0.589670	-2.444935
26.	1.	0.	-2.380691	0.354608	-1.449100
27.	1.	0.	1.095361	-3.743395	-2.564412
28.	1.	0.	-2.484900	-4.219199	-0.269772
29.	1.	0.	0.592297	-0.723067	-3.829538
30.	1.	0.	1.917762	-1.690371	-3.178727
31.	1.	0.	1.394255	-0.201346	-2.366885
32.	1.	0.	-3.675064	-2.947166	1.214532
33.	1.	0.	-4.819552	-1.665405	0.729888
34.	1.	0.	-4.502542	-3.035491	-0.370432
35.	1.	0.	0.266219	-5.735537	-1.800685
36.	1.	0.	-2.406798	-0.299441	0.767646
37.	1.	0.	1.109842	3.519137	3.366339
38.	1.	0.	1.160969	4.686244	2.038029
39.	1.	0.	3.384788	4.185794	2.969613

40.	1.	0.	2.966805	3.581833	0.261282
41.	1.	0.	3.079341	1.405389	1.703828
42.	1.	0.	3.155922	1.734963	3.443598
43.	1.	0.	4.555067	2.084856	2.405864

Table S2. The bioactivity assays of isolated compounds[a]

Compds	(±)-**1**	(+)-**1**	(−)-**1**	**2**	**3**	vancomycin	amphotericin B	oleanolic acid	adriamycin
Staphylococcus epidermidis ATCC 12228	>128	>128	>128	>128	>128	1			
Staphylococcus aureus ATCC 25923	>128	>128	>128	>128	>128	1			
Pseudomonas aeruginosa ATCC 27853	>128	>128	>128	32	128	1			
Bacillus cereus ATCC 14579	>128	>128	>128	128	>128	1			
Escherichia coli ATCC 25922	>128	>128	>128	128	>128	1			
Sarcina lutea ATCC 9341	>128	>128	>128	>128	>128	1			
Candida albicans ATCC 10231	32	16	64	64	>128		0.5		
Candida tropicalis ATCC 20962	>128	128	>128	>128	>128		0.5		
Candida parapsilosis ATCC 22019	>128	>128	>128	>128	>128		0.5		
PTP1B	45.8	17.3	35.5	42.3	>50			4	
A549	>50	>50	>50	>50	>50				0.3
HCT116	>50	>50	>50	>50	>50				0.8
MCF-7	>50	>50	>50	>50	>50				0.2
Hela	>50	>50	>50	>50	>50				0.9

Hep G2	>50	>50	>50	>50	>50							0.4

[a] μg/mL for MIC value of antibacterial or antifungal activity, and μM for IC_{50} value of cytotoxic or PTP1B inhibitory activity.
